# Human gut-derived *B. longum* subsp. *longum* strains protect against aging in a d-galactose-induced aging mouse model

**DOI:** 10.1186/s40168-021-01108-8

**Published:** 2021-09-01

**Authors:** Yue Xiao, Chao Yang, Leilei Yu, Fengwei Tian, Yarong Wu, Jianxin Zhao, Hao Zhang, Ruifu Yang, Wei Chen, Colin Hill, Yujun Cui, Qixiao Zhai

**Affiliations:** 1 State Key Laboratory of Food Science and Technology, Jiangnan University, Wuxi, Jiangsu 214122 People’s Republic of China; 2 State Key Laboratory of Pathogen and Biosecurity, Beijing Institute of Microbiology and Epidemiology, Beijing, 100071 China; 3 School of Food Science and Technology, Jiangnan University, Wuxi, 214122 Jiangsu China; 4 International Joint Research Laboratory for Probiotics At Jiangnan University, Wuxi, 214122 Jiangsu China; 5 National Engineering Research Center for Functional Food, Jiangnan University, Wuxi, 214122 Jiangsu China; 6 (Yangzhou) Institute of Food Biotechnology, Jiangnan University, Yangzhou, 225004 China; 7 Beijing Innovation Centre of Food Nutrition and Human Health, Beijing Technology and Business University (BTBU), Beijing, 100048 People’s Republic of China; 8 School of Microbiology & APC Microbiome Institute, University College Cork, Cork, T12 YN60 Ireland

**Keywords:** Probiotics, *Bifidobacterium longum*, Evolution, Transmission, Aging alleviation, Genome-wide association studies (GWAS), Genomic adaptation, Arginine metabolism, Gut microbiota

## Abstract

**Background:**

Probiotics have been used to regulate the gut microbiota and physiology in various contexts, but their precise mechanisms of action remain unclear.

**Results:**

By population genomic analysis of 418 *Bifidobacterium longum* strains, including 143 newly sequenced in this study, three geographically distinct gene pools/populations, BLAsia1, BLAsia2, and BLothers, were identified. Genes involved in cell wall biosynthesis, particularly peptidoglycan biosynthesis, varied considerably among the core genomes of the different populations, but accessory genes that contributed to the carbohydrate metabolism were significantly distinct. Although active transmission was observed inter-host, inter-country, inter-city, intra-community, and intra-family, a single *B. longum* clone seemed to reside within each individual. A significant negative association was observed between host age and relative abundance of *B. longum*, while there was a strong positive association between host age and strain genotype [e.g., single nucleotide polymorphisms in the arginine biosynthesis pathway]. Further animal experiments performed with the *B. longum* isolates via using a d-galactose-induced aging mouse model supported these associations, in which *B. longum* strains with different genotypes in arginine biosynthesis pathway showed divergent abilities on protecting against host aging possibly via their different abilities to modify the metabolism of gut microbes.

**Conclusions:**

This is the first known example of research on the evolutionary history and transmission of this probiotic species. Our results propose a new mechanistic insight for promoting host longevity via the informed use of specific probiotics or molecules.

Video abstract

**Supplementary Information:**

The online version contains supplementary material available at 10.1186/s40168-021-01108-8.

## Background

Intestinal commensal microbes make critical contributions to human health, and many elicit beneficial effects on the host. *Bifidobacterium* species are pioneer colonizers of the gut and have been associated with various health-promoting effects [1], although the precise modes of action remain largely unknown. The abundances of various *Bifidobacterium* species in the gut vary widely among individuals according to dietary habits [2, 3], age [4, 5], and physiological status [6, 7]. One exception is *Bifidobacterium longum* (*B. longum* subsp. *longum*), which belongs to the human core microbiome [8]. This species accounts for a higher proportion of *Bifidobacterium* species in the gut regardless of host age [1], is distributed broadly across the human lifespan [9], and is among a small subset of gut commensals that can colonize the gut for years [10]. *B. longum* is a potentially important organism with which to evaluate host–microbe coevolution in the gut.

Although extensive probiotic genomics research has been conducted over the past two decades [11–17], most studies have focused on phylogenetic reconstruction and metabolic functions but rarely explored their evolution. Ecological and evolutionary studies of pathogens and gut bacterial commensals have provided tremendous insights into bacterial transmission patterns and drivers of their population differentiation [18–23]. This has provided a framework with which to study and understand the spread and evolutionary mechanisms of probiotic bacteria and could represent a key step toward the informed use of probiotics for the resolution of many health issues.

Increasingly, evidence indicates that specific microbiota-associated health outcomes can be attributed to individual microbial strains [24–26]. The underlying mechanisms may well involve structural variants in the conserved probiotic surface molecules (e.g., biochemical complexity and variability of microbial-associated molecular patterns) on individual strains [27], the presence or absence of phenotype-specific gene elements [28, 29], or currently unexplained factors. Host phenotype or lifestyle may also exert selective pressure on the genotypes of indigenous microbes, as indicated by the host specificity of genes that encode components of vitamin B5 biosynthesis in *Campylobacter* [30], the exclusive presence of porphyran degradation pathways in the gut microbiota of populations that consume seaweed (mostly Japanese) [31], the strong selective pressure on the commensal gastrointestinal species *Bacteroides plebeius* to acquire porphyran degradation capabilities from a marine bacterium via horizontal transfer [32], and the acquisition of antibiotic resistance genes of *A. muciniphila* via recent lateral gene transfer to adapt to the high level of antibacterial gastrointestinal environment in modern lifestyle [33]. Overall, strain-specific genotypes of specific microbes have readily discernible effects associated with the host metabolism and immunity via protective or pathogenic mechanisms. Accordingly, research interests have recently focused on harnessing the cross-talk between the host phenotype and intestinal microbial genotype for therapeutic purposes. Genome-wide association studies (GWAS) have recently been applied to bacterial genomics analyses [30], but few associations have been established between host phenotypes and probiotic bacterial genotypes. We hypothesize that the coevolution of the intestinal microbiota and hosts over millennia has resulted in bacterial-host cross-talk, and this relationship can exert selective pressure on microbial genotypes, while enabling the host to benefit from this microbial genomic adaptation.

Here, we investigate the evolutionary modes and phenotypic associations of health-associated bacteria via a population genomics analysis in which we applied a framework based on ecological theory developed for pathogens or other gut symbionts. We selected *B. longum* as an exemplary representative of host–microbe coevolution in the gut, and because it has been linked to host aging or longevity [34–37]. We conducted a population genomics analysis of *B. longum* with the aim of (1) determining the distribution and transmission of this species both domestically and globally; (2) analyzing and defining the population structure of this bacterium through examining vertical genetic signals disturbed by recombination and identifying population-specific genomics variations; (3) determining associations between host factors (e.g., age, sex, and location) and strain genotypes; and (4) exploring the effects of *B. longum* and its key molecules/pathways on host aging in vivo.

## Methods

### Bacterial strains

In total, 461 *B. longum* strains [147 newly sequenced and 314 publicly available in the National Center for Biotechnology Information (NCBI) database] were preliminarily used in this study. After taxonomic identification via phylogeny reconstruction, only 418 *B. longum* subsp. *longum* strains (143 newly sequenced and 275 publicly available) were used for further analysis (Tables S1 and S2). Ten phylogenetically distant *B. longum* subsp. *longum* strains that were with different single nucleotide polymorphism (SNP) statuses in genes of arginine biosynthesis pathway [five positive strains (with AGT allele at genomic loci 891,726, 891,804, and 891,054): 278(O1), RG4-1 (O2), FJSWXJ10M2 (O3), ZCC2 (O4), and ZCC5 (O5); and five negative strains (with GTC allele): FGSZY16M3 (Y1), FHaNCM25GMM1 (Y2), FSDLZ59M1 (Y3), ZCC12 (Y4), and CCFM752 (Y5)] were chosen for further in vitro assays. Six out of these 10 strains [three positive strains with higher ability to increase arginine level in vitro: O1, O2, and O3; and three negative strains with lower ability to increase arginine level: Y1, Y2, and Y3] were selected for further in vivo animal experiments. The detailed metadata of these 10 strains are highlighted in Table S1. The 6 strains used in the mice have been deposited publicly at the China General Microbiological Culture Collection Center (CGMCC) with respective collection numbers as follows: 278 (CGMCC No. 1.19101), RG4-1 (CGMCC No. 1.19102), FJSWXJ10M2 (CGMCC No.1.18899), FGSZY16M3 (CGMCC No.1.18898), HaNCM25GMM1 (CGMCC No.1.19104), and FSDLZ59M1 (CGMCC No.1.18900). All the other strains obtained in the project will be available upon request. The flow chart of the approaches used for all the included analyses is shown in Figure S1.

### Microbiota analysis

In total, 109 human fecal samples (Table S3 and Figure S2A) were collected in China and stored at − 80 °C until microbiota analysis. Fecal DNA was extracted by using a FastDNA Spin Kit for Soil (catalog number: 116570200, MP Biomedicals, USA) according to the manufacturer’s instructions. For lysis, a repeated bead beating method was used. Samples were placed in Lysing Matrix E tubes (MP Biomedicals) and extracted twice in lysis buffer (4% wt/vol SDS; 500 mmol/L NaCl; 50 mmol/L EDTA; 50 mmol/L Tris·HCl; pH 8) with bead beating at 6.0 m/s for 40 s in a FastPrep-24 instrument (MP Biomedicals). The microbiota analysis pipeline including wet experiments and bioinformatics analysis was conducted according to our previous report [38]. To analyze the genus-level composition of fecal microbiota, the V3–V4 region of the *16S rRNA* gene was amplified by PCR with the isolated fecal bacterial genome as the template. The primers were as follows: forward primer 341F: 5′-CCTAYGGGRBGCASCAG-3′, and reverse primer: 5′-GGACTACNNGGGTATCTAAT-3′. The PCR conditions consisted of an initial denaturalization step of 95℃ for 5 min, followed by 30 cycles of denaturation at 95℃ for 30 s, annealing for 30 s at 52℃, and extension stage at 72℃ for 30 s. At the end of cycling, the reaction was maintained at 72℃ for 10 min. Negative controls using deionized sterile water as the template were included.

Considering our emphasis on the bifidobacterial composition rather than all the analyzable species, sequencing cost, and the weakness of ITS bifidobacterial profiling [39, 40], the 60 kDa chaperonin (*groEL*) gene-based bifidobacterial profiling approach [41], that is cost-effective, accurate in qualification, and evidenced to be effective by multiple studies [38, 42–44], instead of other species-level sequencing methods, was adopted. To distinguish the species within the genus *Bifidobacterium*, *groEL* gene was amplified using the primers Bif-groEL-F (5′-TCCGATTACGAYCGYGAGAAGCT-3′)/Bif-groEL-R (5′- CSGCYTCGGTSGTCAGGAACAG-3′), as previously described [41]. The extracted fecal bacterial genome was set as the template. For the PCR conditions, initial denaturation was at 95℃ for 5 min, with a further 35 cycles at 95℃ for 45 s, 60℃ for 45 s, and 72℃ for 1 min, and then, the reaction was maintained at 72℃ for 10 min. Negative controls using deionized sterile water as the template were included.

The samples were distinguished by a barcode consisting of seven bases that were added to the forward primer 341F or Bif-groEL-F, respectively. For quantification and sequencing, the PCR products (465 bp for the V3-V4 region of the 16S rRNA gene and 480 bp for the *groEL* gene) were excised from a 1.5% agarose gel and purified using the QIAquick Gel Extraction Kit and quantified using the QubitTM dsDNA BR Assay Kit according to the manufacturer’s instructions. Libraries were generated using the TruSeq DNA LT Sample Preparation Kit and sequenced on a Miseq™ sequencer using the MiSeq v3 Reagent Kit (600 cycles-PE) according to the manufacturer’s instructions.

The sequenced reads were analyzed with the QIIME package (Quantitative Insights Into Microbial Ecology). The raw reads were screened following the threshold parameters reported by Mao et al. [45]. Pair-end reads with an overlap of > 10 bp were adopted for assembling. Barcodes and sequencing primers were trimmed from the assembled sequences. The operational taxonomic unit (OTU) was established de novo using uclust with 97% sequence identity cutoff. Any OTUs present in the negative controls were removed from the analysis. The OTUs whose relative abundance was less than 0.005% were removed to decrease the disturbance of the low abundance spurious OTUs. The OTUs of Bif-groEL sequences were taxonomically assigned using the self-built local nucleotide database, and the OTUs of the V3–V4 region were taxonomically assigned using the Ribosomal Database Project (RDP) Naive Bayes classifier [46]. The first sequence in each OTU cluster was selected as the representative sequence. We merged relative abundances of subspecies into a value of species-level relative abundance for *B. catenulatum*, *B. animalis*, *B. longum*, and *B. pseudolongum*, separately. The *16S rRNA* gene sequencing data and *Bifidobacterium* composition data were submitted to the Sequence Read Archive (SRA) under BioProjects PRJNA665348 and PRJNA665364, respectively.

To determine the contribution of host phenotypes to variations in microbiota profiles, transformation‐based redundancy analysis (tb-RDA) was performed using the vegan package of the R software. Detrended correspondence analysis (DCA) was conducted to predetermine the data distribution. The relative abundance of each taxon (genus or bifidobacterial species) was under Hellinger transformation in order to produce valid results in RDA [47], considering that this transformation can accommodate the discrete zero inflated data with many zeros, ensure that the results are comparable across all analyses, produce much more accurate model estimates, and overcome the problems that arise when Euclidean distances are applied to ecological community data without data pre-transformations. Statistical significance was assessed by a permutation test with 1000 random permutations under the full model, which means taking the contributions of all the host phenotypes/factors and the interactions between them into consideration, and by each host phenotype. Permutational multivariate analysis of variance (PERMANOVA) based on Euclidean distance in the vegan package was used to compare group differences in microbiota. Permutation tests using 1000 independent randomizations were used to test for statistically significant differences. Kruskal–Wallis test and/or Mann–Whitney *U* test was performed to compare relative abundances of bacterial taxa by host phenotype.

### *B. longum* isolation and genome sequencing

In total, 148 samples (including the above 109 sequenced samples and additional 39 samples, please see Tables S1 and S3 for detailed information) were used to isolate bifidobacteria by cultivation on deMan, Rogosa, and Sharpe (MRS) agar supplemented with 50 mg/l mupirocin and 0.1% l-cysteine HCl. After incubation at 37 °C for 48 h in an anaerobic chamber (80% N_2_, 10% H_2_, 10% CO_2_), 10 colonies from each sample were picked and subjected to colony-based PCR using species-specific primers [48]. Each identified *B. longum* strain was cultured in MRS broth (with 0.1% l-cysteine HCl) at 37 °C under strictly anaerobic conditions for 16 h before DNA extraction. In total, 147 *B. longum* strains were successfully isolated from 139 samples and used for the following genome sequencing (Table S1). The genomic DNA of each *B. longum* strain was extracted using a rapid bacterial genomic DNA isolation kit (Sangon Biotech Co., Ltd., China). Genome sequencing was performed by using an Illumina HiSeq 2000 sequencer, and a paired-end sequencing library with an average insert size of 350 bp was constructed following the manufacturer’s instructions (Illumina Inc., USA). The maximum read length was set to 150 bp. On average, three GB paired-end raw reads were yielded for each sample. After filtering adaptors and low-quality reads, the obtained clean reads were assembled using SOAPdenovo v2.04 [49], as described previously [50]. The genome data of the newly sequenced strains were submitted to the SRA under BioProject PRJNA665750.

### Single nucleotide polymorphism (SNP) detection, phylogeny, and annotation

The SNPs were recalled for 461 *B. longum* genomes (including 147 newly sequenced strains and 314 publicly available genomes, as shown in Tables S1 and S2 separately) by mapping the assemblies against the reference genome (*B. longum* NCC 2705) using MUMmer [51], as previously described [50], and only bi-allelic SNPs in the core genome were included in the following analysis. After the phylogeny reconstruction (Figure S3), 418 out of these 461 strains were evidenced to belong to *B. longum* subsp. *longum*, 29 belong to *B. longum* subsp. *infantis*, 3 belong to *B. longum* subsp. *suillum*, and 11 belong to *B. longum* subsp. *suis* (Tables S1 and S2). Therefore, the collection of 418 *B. longum* subsp. *longum* strains and its subsets was thus included for the following analyses. Because the number of used assemblies can affect the size of the core genome and the number of detected SNPs [16, 20], different core genome alignments that are constructed by mapping corresponding numbers of assemblies against the reference genome (*B. longum* NCC 2705) were built for analyzing subsets of the data. For example, for phylogenetic reconstruction of all the *B. longum* subsp. *longum* strains, we built core genome of all the 418 strains, and then called SNPs; for GWAS analysis between host factors and *B. longum* genotypes in local panel (only Chinese strains), we built core genome of 144 strains (143 Chinese isolates and reference genome NCC 2705). The sequences of concatenated SNPs were used to construct a phylogenetic tree (neighbor-joining method) using TreeBeST (http://treesoft.sourceforge.net/treebest.shtml), and the tree was visualized by iTOL (https://itol.embl.de/). All of the assemblies were re-annotated using Prokka [52], and then, the Roary software [53] was employed, which takes the annotated results as inputs to identify the pan-genome and the presence or absence of genes for the species (with a minimum BLASTP percentage identity of 90%).

### Recombination rate, population structure, and fixation index (Fst)

The overall r/m value (i.e., the ratio between the numbers of SNPs inside and outside the recombination site) of the 418 *B. longum* subsp. *longum* strains was analyzed by ClonalFrameML [54], taking the RAxML-NG [55] generated maximum likelihood (ML) tree as the input. The phylogenetic tree of 418 strains revealed a radial population structure (Figure S4A) without obvious monophyletic clades, and the bootstrap values of deep branches were often 0, indicating that considerable recombination had occurred. The average r/m value was 4.38. Therefore, we could not infer the population structure and transmission with respect to phylogenetics because recombination had heavily disturbed the vertical genetic signals. Instead, we used fineSTRUCTURE and the output of ChromoPainter to assign individuals to populations with distinct ancestry profiles.

The flow chart of approaches used for population structure analysis is shown in Figure S5. The fineSTRUCTURE software was used to elucidate the population structure of *B. longum* subsp. *longum* based on genome-wide SNPs. We prepared a recombination map file, which is a co-ancestry matrix that contains the number of recombination-derived DNA chunks donated from each donor to each recipient, by specifying a uniform recombination rate per site. fineSTRUCTURE v2 [56] was then run based on the co-ancestry matrix by setting the “-go” parameter for enough iterations of both the burn-in and Markov chain Monte Carlo chain to identify statistically indistinguishable individuals and cluster them. Taking the effects of clonality into consideration, an iterative algorithm was used to successively discard strains with signals of clonal relationship. First, strains with a SNP distance of less than 300 SNPs were randomly removed, and only one representative strain was retained for each clonal group to finally generate a non-redundant genome set of 339 strains (SNP distance of any two strains in the dataset was > 300 SNPs). It should be mentioned that this set of non-redundant genomes was only a transitional data set specifically for population structure analysis. We then ran fineSTRUCTURE using this subset. Forty-three populations were identified in this initial analysis; however, most comprised only two or three strains. These smaller populations disturbed the overall population structure of the species (Figure S6). It has been reported previously that such smaller populations were considered to consist of strains with detectable clonal signal and should be removed for obtaining clear population structure [20, 50]. We therefore randomly removed all but one of the strains in these clonal groups and reran fineSTRUCTURE.

For 295 representative *B. longum* subsp. *longum* strains, SNP sites [57] were used to convert multiple alignments of the core-genome to VCF format. Fst values within and between *B. longum* populations were analyzed using the R package hierfstat [58].

### Population-specific genes/SNPs

The flow chart of approaches used for identification of population-specific genes/SNPs is shown in Figure S7. Gene-based and SNP-based GWAS without correction for population structure were used for identifying distinct genes/variants related to each *B. longum* subsp. *longum* population according to the approach reported previously [19]. Two hundred and ninety-five representative strains retained after two fineSTRUCTURE runs were used for this part of analyses. Although typical GWAS elucidate associations between specific phenotypes and genetic elements while adjusting for population effects, we omitted the control population to search for genomic markers that were varied between different populations, some of which may intrinsically define population structure. Three separate GWAS were conducted to find variable genes/SNPs that were present or absent in the BLAsia1 population alone (BLAsia1 GWAS), the BLAsia2 population alone (BLAsia2 GWAS), and the BLothers population (BLothers GWAS). GWAS pipeline pyseer was used [59], and only variants found in 5–95% of the analyzed strains were used. pyseer was conducted using population arrangements of isolates (BLAsia1/non-BLAsia1, BLAsia2/non-BLAsia2, or BLothers/non-BLothers) as the binary phenotype and the presence/absence of each gene/SNP as the tested genotype. The significance threshold was set using Bonferroni correction with a required *P* value of 0.05/number of variants.

Final sets of population-specific variants (SNPs or genes) were finally obtained by merging the results of the three GWAS. We annotated the biological functions of the SNPs within coding regions and also annotated the genes identified above in terms of functional categories [cluster of orthologous group (COG) term] and pathway data [Kyoto Encyclopedia of Genes and Genomes (KEGG)], using EggNOG [60] with a threshold bit-score of 60, query coverage of 50, and an e-value of 10^−5^. Functional enrichment was conducted as previously reported [19]. In brief, the reference genome NCC2705 harbored the total population-specific SNPs, and 100 of 362 population-specific genes. One-sided Fisher’s exact test was adopted to identify COG functions and KEGG pathways that represented significant deviation from random expectation in the NCC2705 genome. Enrichment analysis for SNPs and genes was performed for 20 COG categories and 200 pathway terms, and the significant threshold was set as a *P* value of 0.01/220 = 4.55 × 10^−5^ using a strict Bonferroni correction.

### Transmission analysis

By setting pairwise SNP distances less than 2500, we defined 31 semi-clonal groups (SCG) with each SCG containing 2–146 isolates for the 418 *B. longum* subsp. *longum* strains. As the number of used assemblies can affect the size of the core genome and the number of detected SNPs, we recalled SNPs for each SCG and used the recombination detection tool (Gubbins) [61] to identify the recombination sites for each SCG. After removing recombination regions, we re-analyzed the pairwise SNP distances between strains of each SCG to identify clonal groups (CGs). A pairwise SNP distance of less than 10 was set as the CG threshold, according to a previous study [62]. Isolates in each CG are the decedents of a common ancestor, and thus are considered as valid candidates to reflect transmission events.

### Phenotype association mapping

The flow chart of approaches used for phenotype association mapping is shown in Figure S8. The pyseer software was used for this analysis [59]. Four phenotypes (province, age, longevous district status, and sex) and three phenotypes (country, age, and sex) were collected for the local panel (the isolated Chinese strains in this study) and the global panel of *B. longum* subsp. *longum* strains (the isolated Chinese strains in this study, and Japanese strains benefiting from detailed record of phenotype information in a previous research [9]), respectively (see Tables S1 and S2 for detailed phenotype information). Among these phenotypes, the term “longevous district status” means whether a strain was isolated from the longevous districts or not. SNP-based and gene-based GWAS were conducted, and a fixed model was adopted. Different core-genome alignments and the resulting SNP matrixes were prepared for each subset of the data. Age served as a continuous phenotype, while the other three phenotypes were in binary format. We used the Mash tool to calculate population structure, and conducted multidimensional scaling (MDS) to determine retained dimensions for the distance matrix. The pairwise distance matrix from Mash was used to adjust the population structure. The SNP matrix and the gene presence/absence table were used as a genotype matrix. The genomic variants were filtered to ensure that they appeared in >1% and <99% of samples. The significance threshold was set using Bonferroni correction with a required *P* value of 0.05/number of variants. Visualization of the data from GWAS was achieved by drawing Manhattan plots using the qqman package in R. We also used RDA analysis and PERMANOVA (based on Euclidean distance) to validate the above phenotype association results, and calculated the relative importance of these phenotypes for explaining the genomic variations. It should be mentioned that the relative importance of host phenotypes can only be accessed (via RDA and PERMANOVA analyses) when the phenotypes are shared among all the analyzed strains; as long as a strain lacks any phenotype, the strain should be excluded from the analysis. Therefore, the abovementioned two panels were adopted for the phenotype association mapping, including the local panel (Chinese strains with shared four phenotypes) and the global panel (Chinese strains and Japanese strains with shared three phenotypes). For other entries apart from Chinese and Japanese strains in the total dataset of 418 strains, no phenotype or only country phenotype was available, and thus, these entries were precluded from the analysis.

### Animal experiments

#### Strain selection and in vitro assays for measuring the ability of a strain to alter arginine levels

Ten *B. longum* subsp. *longum* strains with divergent SNP statuses [five strains for each genotype (AGT or GTC in the genomic loci 891,726, 891,804, and 891,054)] in the arginine biosynthesis pathway were preliminarily selected, and their ability to alter the arginine level of the culture supernatant was determined. Briefly, each strain was inoculated into MRS broth with 0.1% l-cysteine HCl, and culture medium without inoculation served as a blank. After cultivation to early stationary phase (OD_600_ = 5.0; the accuracy of the bacterial cell number was determined by plate counting), the culture was centrifuged to remove bacterial cells, and after precipitating protein by trichloroacetic acid (CAS number 76–03-9, Sinopharm Chemical Reagent Co., Ltd., China), the supernatant (including the medium blank) was directly used for determining the arginine level using an amino acid analyzer (L-8900, Hitachi, Japan). Alterations in the arginine level after bacterial cultivation were measured and expressed as ΔArg. Three pairs of *B. longum* subsp. *longum* strains (each pair comprised two phylogenetically close strains with different abilities to adjust arginine metabolism) were finally selected out of the 10 strains to conduct animal experiments.

#### Animals and experimental design

Eight-week-old male C57black/6 J mice used in this study were purchased from the Shanghai Laboratory Animal Center (Shanghai, China). Animal care and study protocols were approved by the Ethics Committee of Jiangnan University, China (JN. No20181215b1000130[269]). All the applicable institutional and national guidelines for the care and use of animals were followed. The mice were kept in the mouse facility at the Laboratory Animal Center of the Department of Food Science and Technology, Jiangnan University, Wuxi, China, on a 12-h light/dark cycle in a temperature- (22 °C ± 1 °C) and humidity-controlled (55% ± 10%) room.

Mice were assigned to different experimental groups (*n* = 9 for each group). The aging model was generated by administering d-galactose (CAS number 59–23-4, Sinopharm Chemical Reagent Co., Ltd., China) via subcutaneous injection at a dose of 1000 mg/kg BW/d according to our preliminary results (data not shown) and a previous study [63]. Mice in the control group received sterile saline via subcutaneous injection, while the other groups were treated with saline-based d-galactose. For arginine supplementation, arginine was added to the normal mouse chow diet to ensure a dose of 0.4 mg/g BW/d according to a previous report [64]. *B. longum* strains were freshly cultured in MRS broth, then resuspended in sterile saline, each day, and plate counts were conducted to ensure a gavage dose of 10^8^–10^9^ CFU/d for each mouse. Related to the daily preparation of strains over 9 weeks, F2 cultures for each strain were used and grown from the same original cryo-stock to avoid possible genetic drift of strains. For the control group, an equal volume of sterile saline was administered. The body weight and feed intake of all of the mice were recorded daily, and the doses of arginine and d-galactose were adjusted correspondingly.

#### Behavior test

For the behavior tests, the testing room was fitted with an adjustable dimmer light within 280 lx, and mice were transferred into the room at least 30 min before testing. Testing equipment was cleaned regularly using 70% ethanol between events to avoid olfactory cuing. The open-field test, Morris water maze (MWM) test, step-through test, and Y-maze test were performed (see the Supplementary materials for detailed experimental settings and procedures).

#### Antioxidative parameters

One day after all of the behavior tests had been completed, the mice were euthanized. Tissues were collected immediately and stored at − 80 °C for measuring the antioxidative parameters within 1 week. The levels of MDA and the activities of GSH-Px, SOD, and CAT in the liver and brain were evaluated according to the instructions of the manufacturer using assay kits (Jiancheng Bioengineering Institute, Nanjing, China).

### Occurrence of ingested *B. longum* strains

The detailed protocol for detecting the occurrence of ingested *B. longum* strains is described in the supplementary methods.

#### Metabolomics of the gut microbiota

The detailed protocol for fecal metabolomics analysis is described in the Supplementary methods.

#### Statistical analysis

All the data from the animal experiments were confirmed to have a normal distribution by the Kolmogorov–Smirnov (KS) normality test and were analyzed by one-way ANOVA. The scaled data were used for principal component analysis (PCA) via prcomp in the R software. Metabolites showing different concentrations for each pairwise comparison were identified with ANOVA [false discovery rate (FDR)-adjusted *P* < 0.05] and orthogonal partial least squares discriminant analysis [OPLS-DA, variable important in projection (VIP) > 1]. Cross-validation with 200 permutations was conducted to avoid over-fitting by the OPLS-DA analysis. The PLS-DA plot based on all the tested metabolic features of the nine experimental groups was analyzed and plotted using the R package “mixOmics”. The OPLS-DA analysis was conducted via R package “ropls”. The pathway enrichment analysis based on metabolites with known KEGG IDs was performed through the MBrole online tool against the complete KEGG database.

## Results

### Effect of environmental factors on the human gut microbiota and bifidobacteria

Although some studies have focused on the characteristics of the human gut microbiota at the genus, species, and even strain levels [65–69], the distribution of gut bacterial genera and *Bifidobacterium* species, and their relationships with host factors remain largely unknown for Chinese populations. Here, we sequenced 109 fecal samples from subjects of both sexes who ranged in age from birth to 105 years and resided in longevous districts (districts with high ratios of centenarians) or normal areas of 16 provinces (or municipalities) in China.

**Fig. 1:**
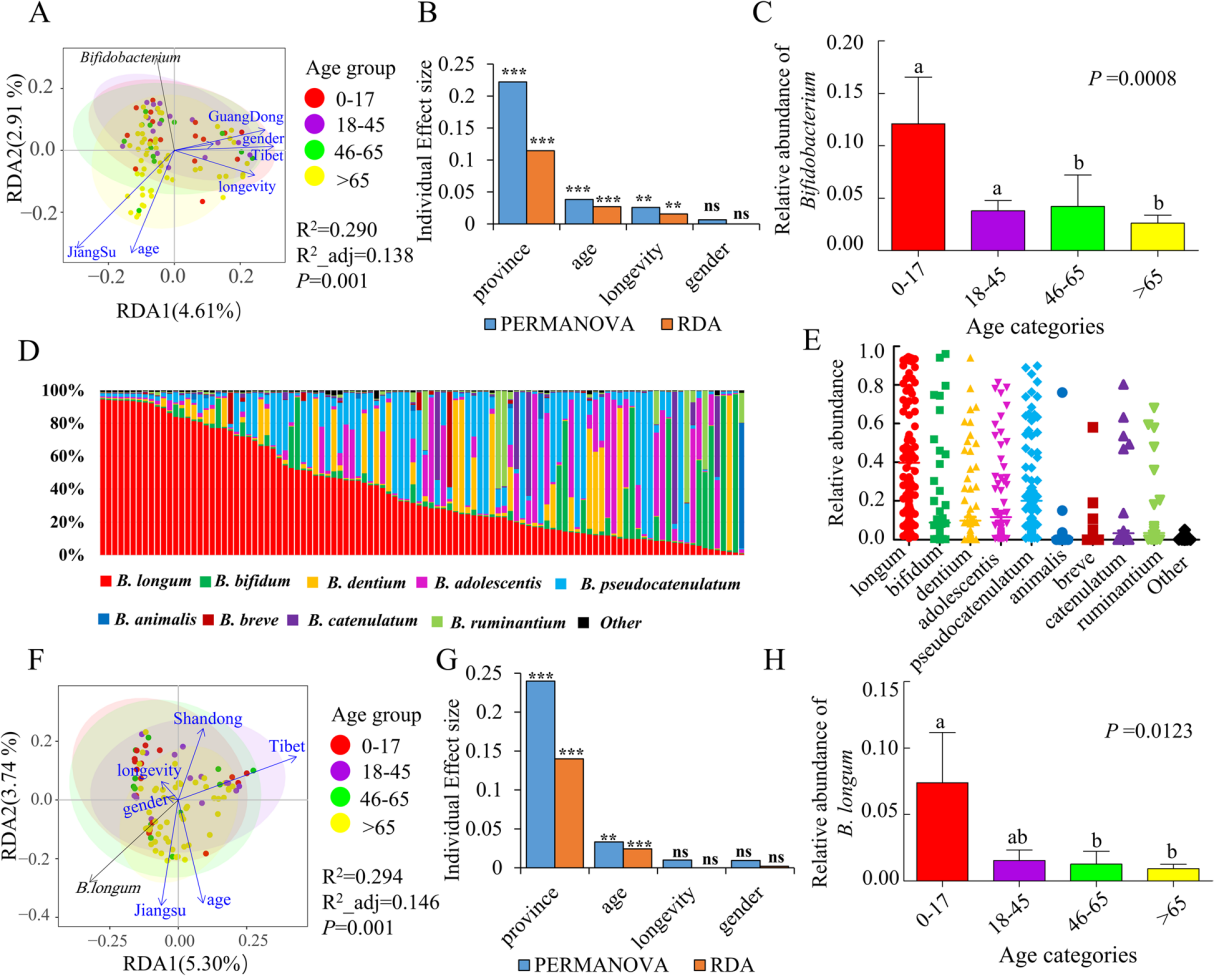
*Bifidobacterium*, particularly *B. longum*, was prevalent and dominant in the guts of subjects in a Chinese cohort, and the relative abundances of this genus and species were significantly associated with host age. **A** Triplot of a distance-based redundancy analysis (db-RDA) of the microbiota composition at the genus level relative to province, age, longevous district status, and sex. **B** Individual effect sizes of the gut microbiota covariates based on genus-level db-RDA and PERMANOVA analyses. **C** Comparisons of the *Bifidobacterium* relative abundance between age segments. The overall *P* value was calculated by Kruskal–Wallis test, while pairwise comparisons were conducted by Mann–Whitney *U* test. Significant differences (*P* < 0.05) between the age categories are indicated with different letters (a and b) above the bars. **D** *Bifidobacterium* species-level composition of the gut microbiota. Each column represents a sample. The stacked bars have been sorted according to the decreasing occurrence of *B. longum*. **E** Abundance of each bifidobacterial species relative to the total *Bifidobacterium*. Each dot represents a sample. **F** Triplot of a db-RDA of the microbiota composition at the *Bifidobacterium* species level relative to province, age, longevous district status, and sex. **G** Individual effect sizes of the gut microbiota covariates based on the *Bifidobacterium* species-level db-RDA and PERMANOVA analyses. **H** Comparisons of the *B. longum* relative abundance between age segments. The overall *P* value was calculated by Kruskal–Wallis test, while pairwise comparisons were conducted by Mann–Whitney *U* test. Significant differences (*P* < 0.05) between the age categories are indicated with different letters (a and b) above the bars. Among these analyzed phenotypes, the term “longevity/longevous district status” means whether a strain was isolated from the longevous districts or not. *P < 0.05, **P < 0.01, ***P < 0.001

The bacterial compositions of the samples were sequenced and analyzed. As shown in Figure S2B and Table S4, *Bifidobacterium* was among the 18 core genera (accounting for 78.34% of the total sequences) within the cohort and was ranked sixth in terms of detected relative abundance (accounting for 4.26% of the total sequences) among a variety of gut genera, although the relative abundances of this species showed great individuality. To evaluate the individual contributions of the isolation location, age, longevous district status (the term means whether a strain was isolated from the longevous districts or not), and sex on the microbiota composition, we performed a distance-based redundancy analysis (db-RDA) and Adonis PERMANOVA analysis. The results showed that these additional covariates explained at least 13.8% of the variation in fecal microbiota (13.8% for db-RDA and 29.2% for PERMANOVA; Fig. 1A and B). However, the only statistically significant individual factors were province (db-RDA 11.4% and PERMANOVA 22.2%), age (db-RDA 2.7% and PERMANOVA 3.8%), and longevous region status (db-RDA 1.6% and PERMANOVA 2.6%). Among the tested genera, it is notable that *Bifidobacterium* was the top 2 member for which its relative abundance was markedly different among age categories (*P* = 0.0008 for Kruskal–Wallis test; Fig. 1C and Figure S2C), showing a decrease trend with increase of age. For pairwise comparisons between age categories, age group 0–17 and age group 18–45 respectively showed marked difference in *Bifidobacterium* relative abundance compared with either of the other two age groups (46–65 and > 65; *P* < 0.05 for Mann–Whitney *U* test). Apart from host age, relative abundances of some genera also varied significantly by the isolation location, longevous district status, or host sex (Table S5).

We then tested the composition of *Bifidobacterium* species in the samples based on *groEL* gene-based bifidobacterial profiling. *B. longum* was present in every individual and was the most dominant species (36.08% in total for the sequences tested, rank: 1st) across both sexes, all age groups, and different isolation locations (Fig. 1D and E, and Table S6). Similarly, four phenotypes accounted for at least 14.6% of the fecal bifidobacterial variation (14.6% for RDA and 29.3% for PERMANOVA; Fig. 1F and G). The isolation location (RDA 14.0% and PERMANOVA 24.0%) was the first contributor to the variation, followed by age (RDA 2.4% and PERMANOVA 3.3%), whereas the composition was not affected by longevous region status (*P* = 0.407) or sex (*P* = 0.217). The abundances of individual bifidobacterial species relative to the total *Bifidobacterium* were normalized by the relative abundance of the genus *Bifidobacterium* in each sample (Figure S2D), and a generated abundance matrix was used to conduct comparisons of biomasses of individual *Bifidobacterium* species by each of host factors. Notably, *B. longum* was among the tested species for which the relative abundances showed significant differences among age segments with an obvious decrease along age axis (*P* = 0.0123 for Kruskal–Wallis test; Fig. 1H and Figure S2E). Significant higher level of *B. longum* relative abundance was observed in the age group of 0–17 compared with either of two age groups (46–65 and > 65; *P* < 0.05 for Mann–Whitney *U* test), while age group of 18–45 showed no marked difference in *B. longum* relative abundance compared with each of the other three age groups. In addition, significant differences in relative abundances of individual bifidobacterial species were also observed for the other host phenotypes except host sex (Table S5).

These results suggest that geography and host phenotype have significant effects on the microbiota community structure at both the genus and bifidobacterial species levels. *Bifidobacterium*, particularly *B. longum*, was prevalent and dominant within the studied cohort and showed a significant association with host age. Therefore, *B. longum* can be considered to be a ubiquitous gut microbe that interacts actively with the host, and its genotype may well serve as a candidate molecular marker of evolutionary events.

### Three geographically distinct *B. longum* gene pools

**Fig. 2:**
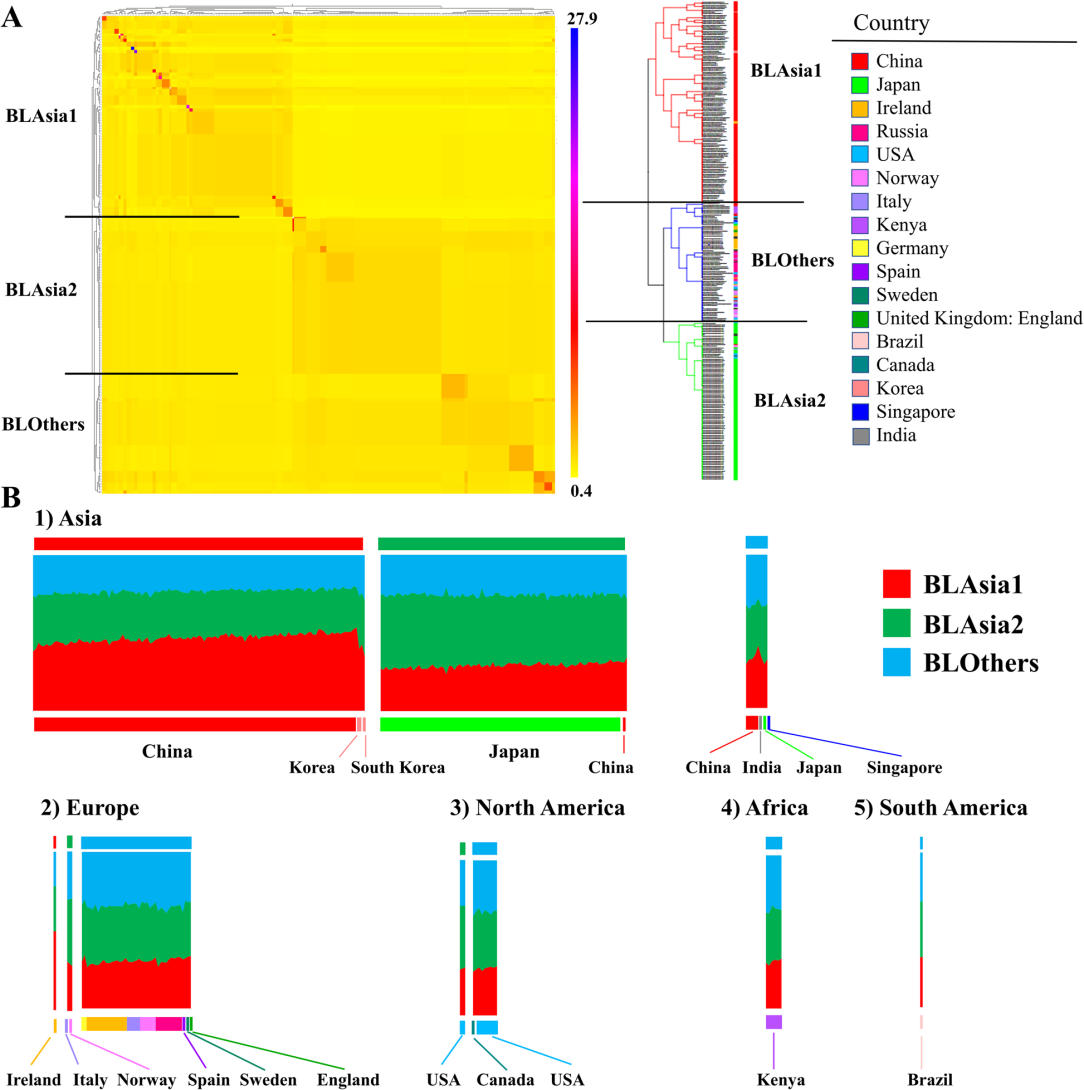
Formation of three geography-related populations and evolution driven mainly by admixture within each population. **A** *B. longum* population structure revealed by FineSTRUCTURE analysis. The color of each cell in the co-ancestry matrix represents the anticipated number of DNA chunks imported from a donor strain (column) to a recipient strain (row). The color bar near the tree represents the geographical locations where the strains were sampled. **B** Genomic compositions of *B. longum* strains inferred by chromosome painting. Each vertical bar represents one strain, and the bars are sorted by geographical location. The color of each bar represents the contribution by each of the three populations to the core genome of that strain. The color bar at the bottom indicates the sampling locations. Two hundred and ninety-five representative strains retained after FineSTRUCTURE runs were used for this part of analyses

To examine the evolution and transmission mode of *B. longum*, we isolated and sequenced 143 *B. longum* subsp. *longum* strains from 100 out of the abovementioned 109 Chinese fecal samples and additional 39 samples in our laboratory collection, and combined these with 275 publicly available genomes of the subspecies, resulting in a dataset of 418 genome sequences from four continents and 17 countries that were isolated primarily from the gut (375/397; 21 genomes with missing niche values; Tables S1 and S2, Fig. 2A, and Figure S4A). A single nucleotide polymorphism (SNP) analysis of the core genome indicated comparable genetic diversity among strains across continents and countries, with the exception of strains from Japan that exhibited relatively greater diversity (Figure S4B and C), as determined by pairwise SNP distances. This relatively greater diversity of Japanese strains seemed to be not correlated with the high number of *B. longum* strains isolated in Japan, since China with the highest number of included genomes (197) showed the middle rank of genomic diversity, while Italy with only 7 sequenced genomes ranked the second.

Our fineSTRUCTURE analysis identified three populations, each containing 124, 97, and 74 representative members, and defined them as BLAsia1, BLAsia2, and BLothers, respectively (Fig. 2A and Figure S4A). The majority of isolates in BLAsia1 were from China (96.8%, 120/124), with three strains from Korea and one strain from Ireland. BLAsia2 predominantly included Japanese isolates (92.8%, 90/97), with two strains from the USA, one strain from Norway, one strain from China, one strain from Italy, and two strains from unknown areas. BLothers included isolates from diverse geographical locations, such as Kenya (Africa), Ireland (Europe), and the USA (North America), which could likely be categorized further into subpopulations if additional strains were available. Co-ancestry plots (Fig. 2A) and fixation index (Fst) values (Figure S4D) revealed closer genetic configurations between BLAsia2 and BLothers, whereas BLAsia1 was more distinct. These distinct populations exhibited comparable genetic diversity in terms of pairwise SNPs (Figure S4D and E).

In the chromosome painting analysis, the strains from each population received a large proportion of their palettes from within their own populations, confirming their differentiation from the other populations (Fig. 2B and Figure S4F). The formation of differentiated populations suggested an admixture within each gene pool. The palettes also provide evidence of genetic mixtures between populations within countries. The Chinese isolates (*n* = 6) that were not assigned to BLAsia1 had higher ratios of components associated with BLAsia2 and BLothers (Fig. 2B and Figure S4F), and at least two populations existed within some countries, including China, Japan, the USA, Ireland, Italy, and Norway, with their palettes representing higher proportions of their respective populations, which is consistent with transmission and recent genetic exchange.

### Population-specific genomic loci suggested significant variations in cell wall biosynthesis and carbohydrate metabolism among populations

To investigate the genetic basis for distinguishing distinct *B. longum* populations, we used a GWAS approach to systematically screen for particular SNPs in the core genome and specific genes in the accessory genome that were present differentially in each population.

**Fig. 3:**
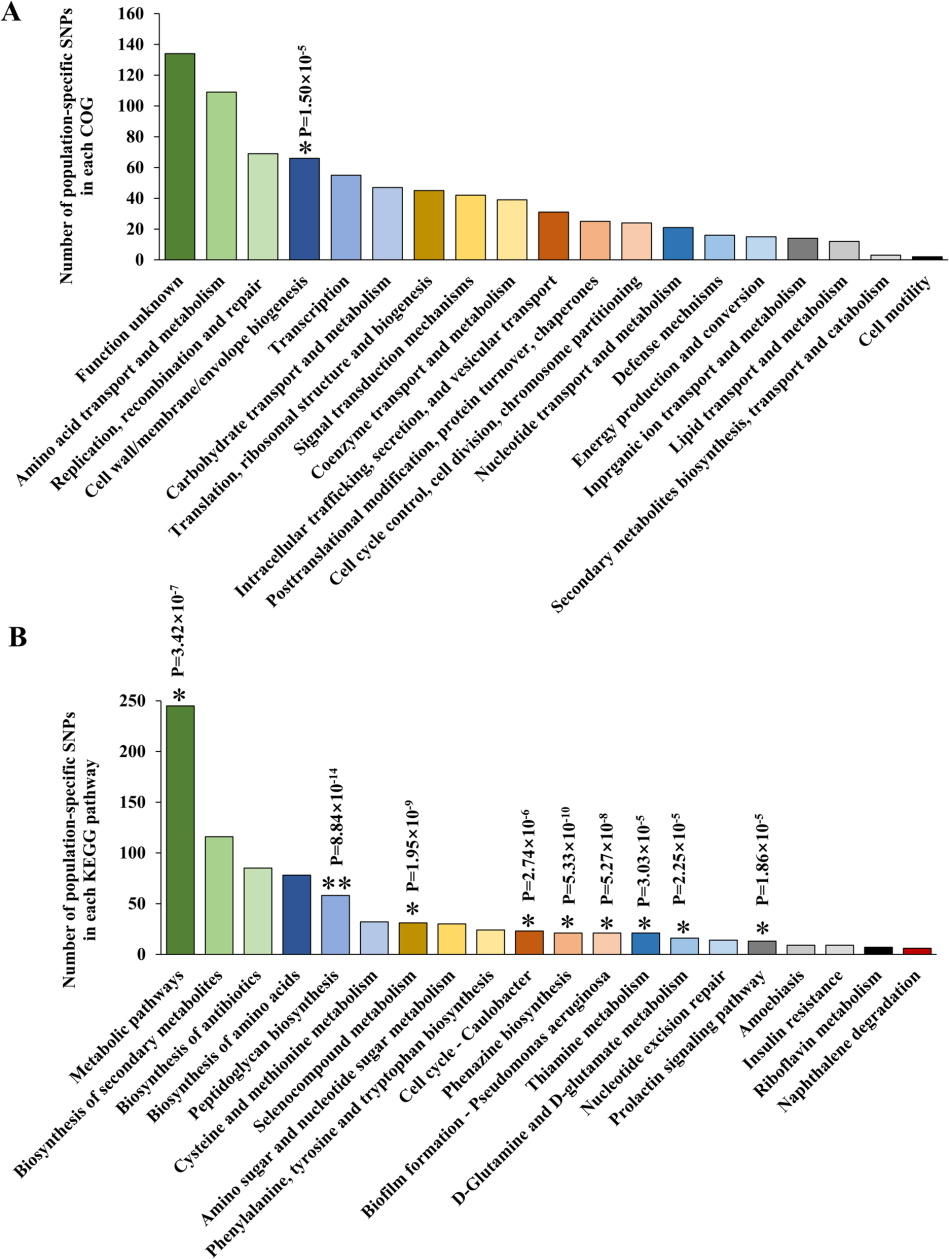
Population-specific genetic profiles. COG (**A**) and KEGG pathway analyses (**B**) of population-specific SNPs in the core genome. Functional or pathway enrichment was evaluated using a one-sided Fisher’s exact test to identify significant terms against random expectation based on the reference genome NCC2705. The enriched terms are marked with asterisks. *P < 10^−5^, **P < 10^−10^. Two hundred and ninety-five representative strains retained after fineSTRUCTURE runs were used for this part of analyses

Regarding the SNPs in the core genome, cell wall biosynthesis, particularly peptidoglycan biosynthesis, was the most significant discriminant among the three populations as evidenced by both COG enrichment and KEGG analyses (Fig. 3A and B, Tables S9 and S10). SNPs were accumulated densely in individual genes and frequently involved non-synonymous loci, and these alterations could well have direct effects on strain phenotypes such as gut fitness.

**Fig. 4:**
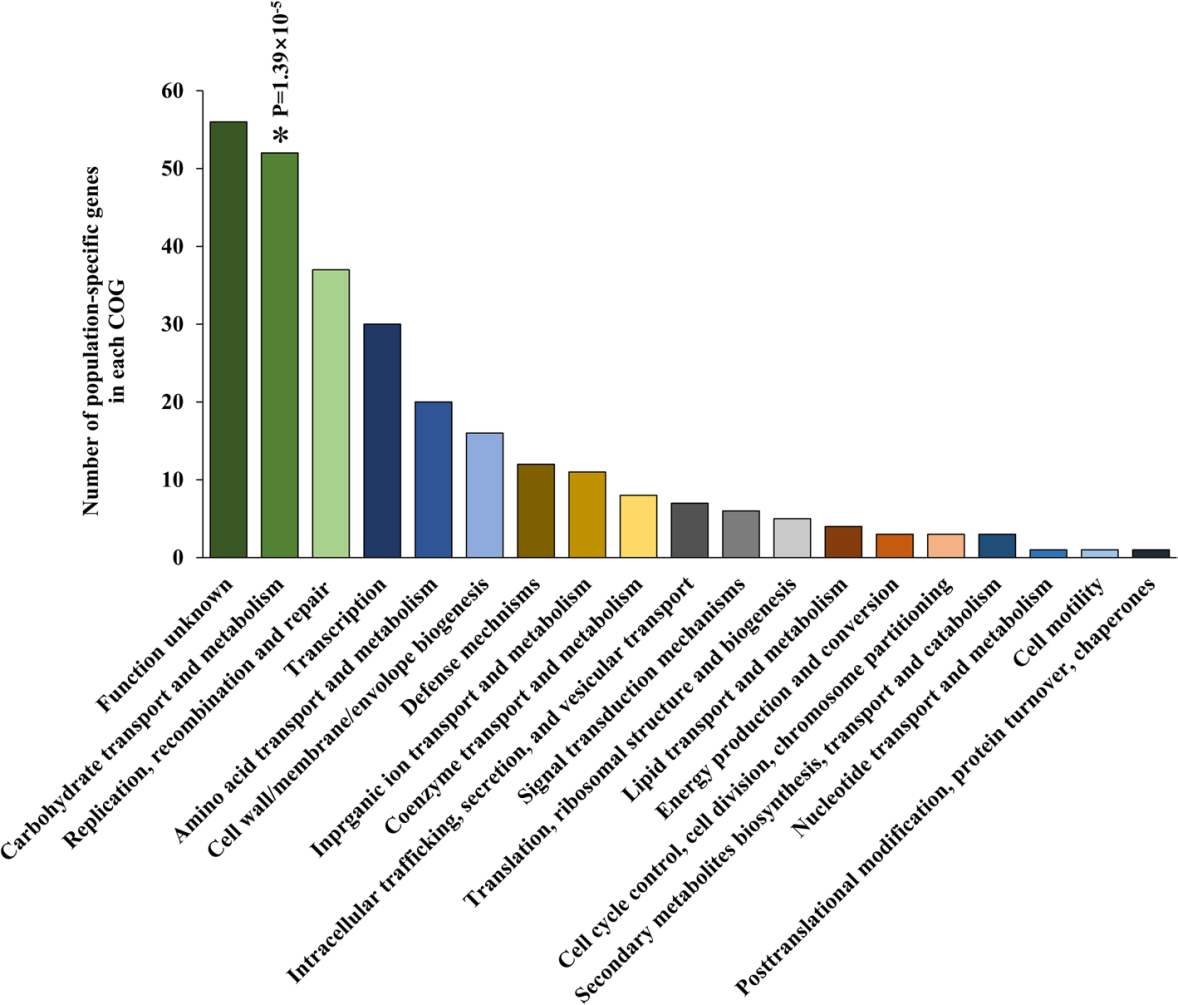
Population-specific genetic profiles. COG analysis of population-specific accessory genes. Functional enrichment was evaluated using a one-sided Fisher’s exact test to identify significant terms against random expectation based on the reference genome NCC2705. The enriched terms are marked with asterisks. *P < 10−5, **P < 10−10. Two hundred and ninety-five representative strains retained after fineSTRUCTURE runs were used for this part of analyses

In the accessory genome, only the carbohydrate transport and metabolism category were identified as significant in COG analysis (Table S11 and Fig. 4, one-sided Fisher test: *P* = 1.39 × 10^−5^), and no significant KEGG pathways were detected (Table S12). Generally, genes involved in arabinose and lactose transport and metabolism varied markedly between the three populations in both the presence or absence of genes and the paralog compositions of specific genes (Figure S10). In addition, the paralog sequences of these genes were also highly dissimilar (Figures S11, S12 and S13). For the detailed description on the related results of this subsection, see the supplementary results.

### *B. longum* undergoes active transmission within families and communities, via inter-city, and inter-country routes, and across different hosts

The frequent transmission of pathogens (e.g., *Helicobacter pylori*) vertically from mother to infant and between individuals in close proximity has been well established [18]. Human activities, such as migration, have been reported to drive the global and regional dissemination of pathogenic microbes [15, 16]. Although the accumulated evidence based on strain resolution also indicates the existence of vertical transmission of gut symbionts, including *Bifidobacterium* species [70, 71], the effects of geography and proximity on the transmission of these microbes remain largely unknown.

As shown in Table S13, several geographic levels of transmission have been identified in terms of inter-country spread (P29 and CG8), inter-provincial spread (CG3, P6, CG4, CG5, P3, and P4), and transmission between different cities within the same province (CG4). Overall, our analysis indicates the existence of inter-country, inter-provincial, and inter-city transmission of *B. longum* strains, particularly in China, which suggests a possible association with population migration.

Interestingly, we also observed transmission between individuals in close proximity. An analysis of 16 strains from 16 residents of a home for the elderly in Wuxi, Jiangsu, yielded five distinct clonal groups (CG6, CG11, P8, P9, and P26), which suggested strain transmission within the community. Transmission between family members has also been identified (CG12), and we also observed transmission of *B. longum* strains between a human and chicken (P10) in the same household.

The isolation of multiple strains within an individual sample was also of interest. We observed that strains isolated from the same individual were clonally related (CG1, CG2, P5, P10, P17, P21, P22, P23, and P32 for nine different human subjects) with a median pairwise SNP value of 2, consistent with a single colonisation event in which an individual subject was colonised by a unique clone. For the detailed description on the related results of this subsection, see the supplementary results.

### Genome-wide association identifies arginine biosynthesis as a host age-associated factor in *B. longum*

GWAS can identify the causal genetic factors that underlie important phenotypes but are rarely applied to the analyses of gut symbionts. Here, we applied GWAS of both core and accessory genome variations to identify links between the host phenotypes and *B. longum* genotypes. We also used RDA and PERMANOVA analyses to re-confirm the significant variations identified in the GWAS and to access the individual contributions of phenotypes to the overall bacterial genotypes.

**Fig. 5:**
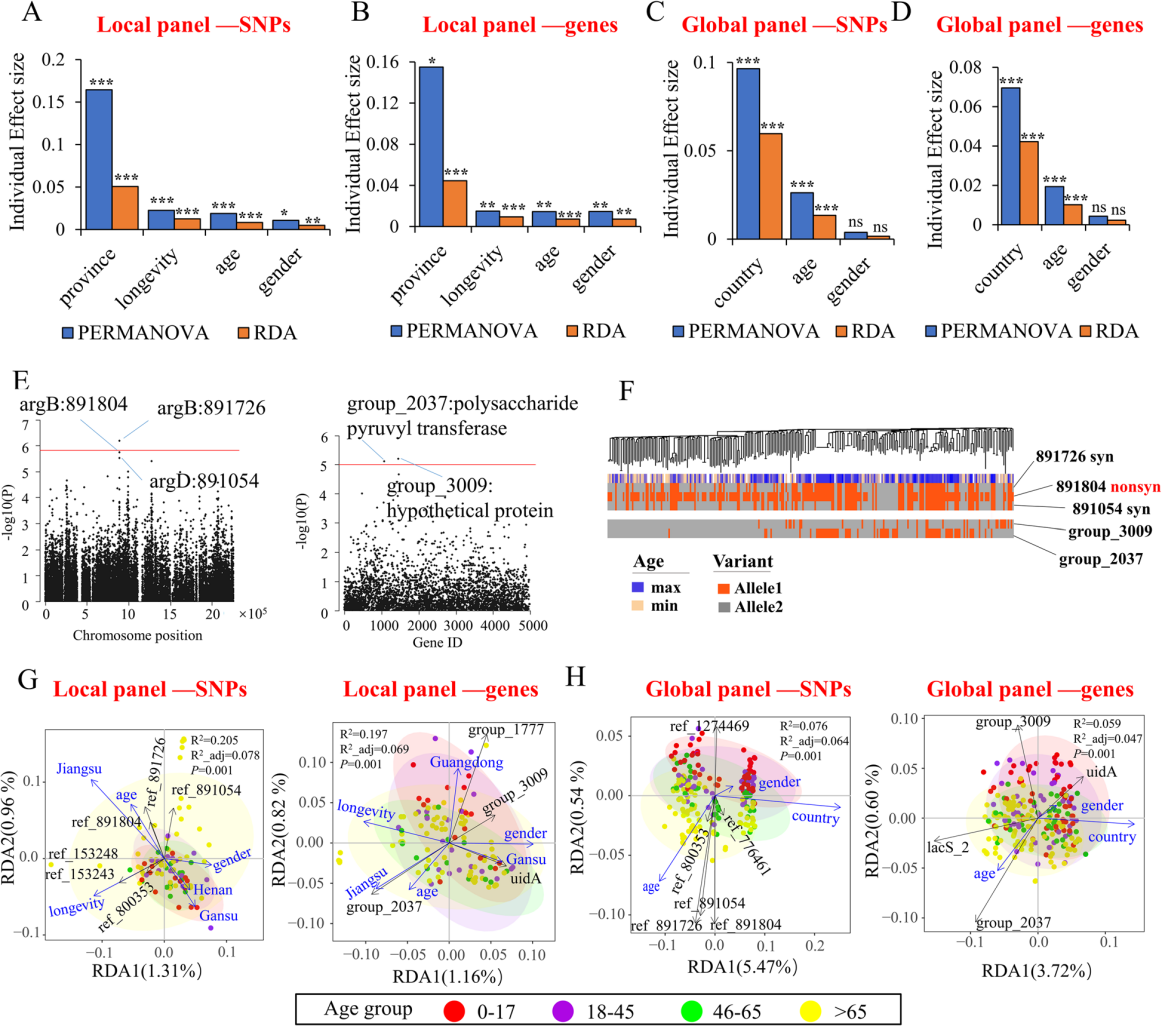
Environmental factors significantly discriminated the overall genotypes of *B. longum*, and arginine biosynthesis of *B. longum* was identified as a potential host age-associated factor. **A** Individual effect sizes of genome covariates (isolated province, longevous district status, age, and sex) determined by SNP-level db-RDA and PERMANOVA analysis of a local panel (Chinese strains). **B** Individual effect sizes of genome covariates based on a gene-level db-RDA and PERMANOVA analysis of a local panel. **C** Individual effect sizes of genome covariates based on SNP-level db-RDA and PERMANOVA analysis of a global panel (Chinese and Japanese strains). **D** Individual effect sizes of genome covariates based on gene-level db-RDA and PERMANOVA analysis of a global panel. **E** Manhattan plot of GWAS results for the associations of age with the genomic profiles of *B. longum* based on core genome SNPs (left) and genes (right). The significance threshold is indicated by a horizontal red line and was defined using Bonferroni correction with a required *P*-value of 0.05/number of tested variants. **F** Distribution of significant variants according to phylogeny and host age. **G**, **H** Triplots of the db-RDA analysis of genomic composition relative to the host province (or country), age, longevous district status, and sex. Different datasets were used for these analyses. **G** (left) SNP profiles of the local panel; **G** (right) gene profiles of the local panel; **H** (left) SNP profiles of the global panel, and **H** (right) gene profiles of the global panel. Among these analyzed phenotypes, the term “longevity/longevous district status” means whether a strain was isolated from the longevous districts or not. *P < 0.05, **P < 0.01, ***P < 0.001

In the local panel, all factors had a significant effect on the *B. longum* genotypes (Fig. 5A and B). Province was the most important discriminant for both SNPs (16.4% and 5.1% of overall genetic variations according to PERMANOVA and RDA, respectively, Fig. 5A) and genes (15.5% and 4.5%, respectively, Fig. 5B), followed by longevous district status, age, and sex. In the global panel, country was the most important discriminant, with individual effect sizes of 6.0–9.6% for the core genome and 4.2–7.0% for the accessory genome (Fig. 5C and D). The genotypes were also markedly affected by host age, but not by sex. Overall, the results implied that all examined host phenotypes other than sex could significantly affect *B. longum* genotypes, although geography was the primary contributor.

The GWAS revealed one SNP and two genes in *B. longum* that were strongly associated with host age (Fig. 5E). Group_2037, which encodes a polysaccharide pyruvyl transferase, was more prevalent in elderly subjects, whereas group_3009 was predominant among young people (Fig. 5F). The arrangement of these two genes was consistent with the age distribution but both were frequently absent from the Japanese lineage (Fig. 5F). Interestingly, a SNP locus (synonymous mutation SNP_891726) located in *argB* was the variant most significantly associated with age, followed by two other adjacent loci (non-synonymous mutation 891,804 and synonymous mutation 891,054) in *argB* and *argD*, which were slightly below the significance threshold. Both *argB* and *argD* encode enzymes in the bacterial arginine biosynthesis pathway, and the distributions of the three loci among *B. longum* strains were highly consistent with the host age distribution (Fig. 5F). RDA analysis confirmed this association, as the variable axes of these loci were highly consistent with the axis of age in both data panels (Fig. 5G and H). Moreover, genomic profiles stratified by four defined age groups were arranged along the age axis in age-ascending order, particularly for SNPs in the global panel (Fig. 5G). This pattern further indicated the strong association of *B. longum* genotypes with host age. No significant variations were detected with respect to the other phenotypes (Figure S14A–G). Regarding the country phenotype, however, we observed a more frequent distribution of *lacS_2* in Chinese strains (118/144), whereas this gene was largely absent from Japanese strains (17/121), which was consistent with the results presented above for the analysis of population-specific loci. For the phenotype of longevous district status, two loci in *dnaE* nearly reached the significance threshold, and this association was also confirmed by the RDA axis (Fig. 5G).

Taken together, the data demonstrate that factors such as geography and host age are significant discriminants of the overall *B. longum* genotype. Although geography might be the primary factor for population differentiation, host age may also be a strong contributor in terms of the distribution of specific variations, and arginine biosynthesis appears to be a host age-associated factor in *B. longum*.

### *B. longum* strains with different arginine metabolism-related genotypes represent divergent alleviation against host aging

The significant reverse associations between host age and *B. longum* relative abundance and the strong link between host age and strain genotype (particularly SNPs in arginine biosynthesis-related genes) suggest the potential effect of *B. longum* and its key pathways on host aging.

**Fig. 6:**
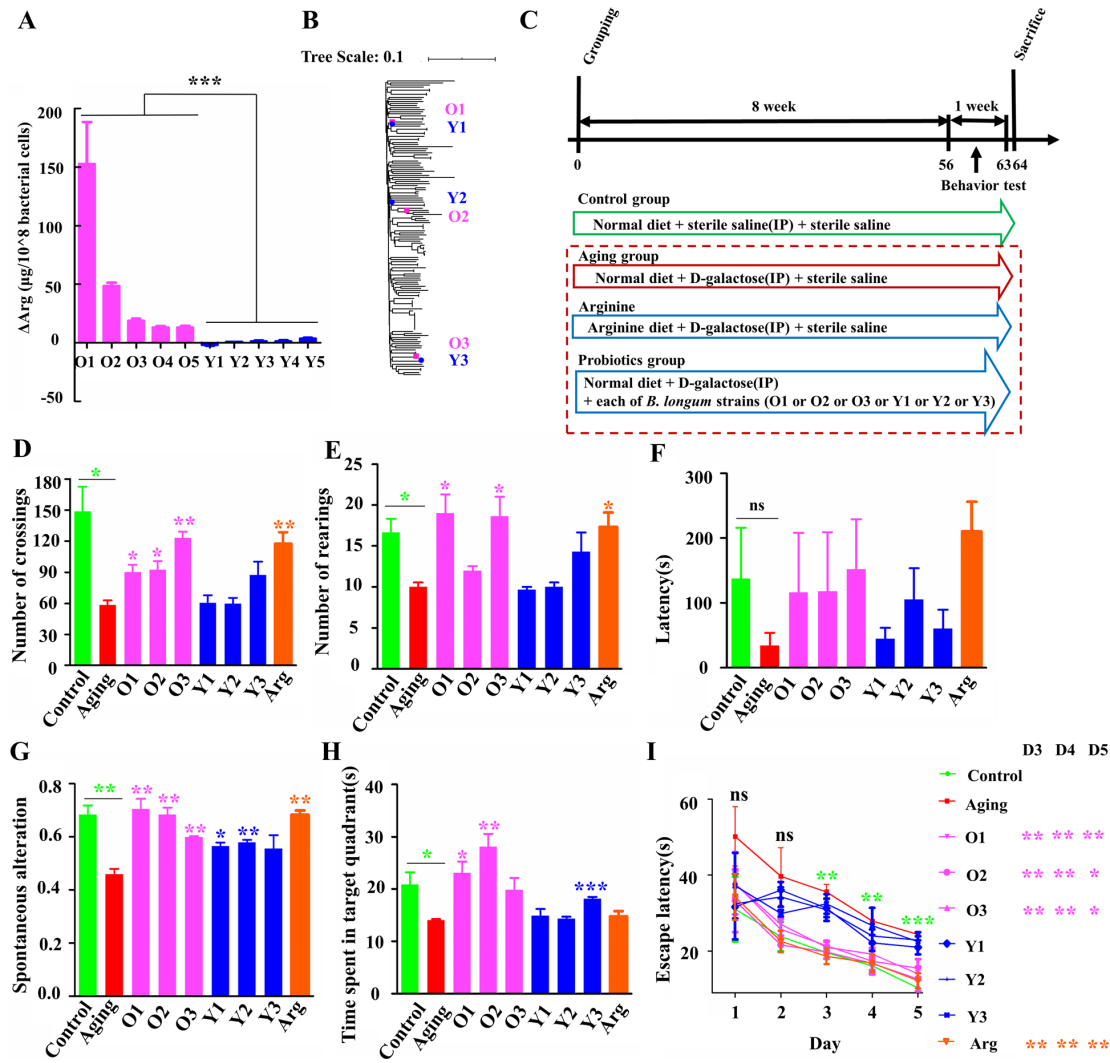
*B. longum* strains with specific genotypes can significantly reverse behavioral changes in aging mice. **A** Ability of *B. longum* strains with different SNP statuses [five positive strains (with AGT allele at genomic loci 891,726, 891,804, and 891,054): 278(O1), RG4-1 (O2), FJSWXJ10M2 (O3), ZCC2 (O4), and ZCC5 (O5); and five negative strains (with GTC allele): FGSZY16M3 (Y1), FHaNCM25GMM1 (Y2), FSDLZ59M1 (Y3), ZCC12 (Y4), and CCFM752 (Y5)] in arginine biosynthesis pathway genes to increase arginine levels in culture supernatants. **B** Locations of six selected phylogenetically distant strains [three positive strains with higher ability to increase arginine level in vitro: O1, O2, and O3; and three negative strains with lower ability to increase arginine level: Y1, Y2, and Y3] used in the mice in a neighbor-joining tree. **C** Diagram of the experimental design. Please see the “Methods” section for additional details. Behavioral parameters. Open-field test (D and E), step-through test (F), Y-maze (G), and Morris water maze (H and I). The normal distribution of all data was confirmed using the Kolmogorov–Smirnov (KS) normality test. All data were analyzed using one-way ANOVA and are presented as the means ± standard errors of the means; *n* ≥ 3 for each group. Arg, Arginine group. Statistical significance was calculated for the comparisons between the aging group and the control group (*s in green color), and for the comparisons between the aging group and each of strain treatment groups (*s in the other colors). *P < 0.05, **P < 0.01, ***P < 0.001

The results from in vitro assays indicated that *B. longum* subsp. *longum* strains with the AGT allele in the genes of arginine biosynthesis pathway (positive strains) exhibited a significantly improved ability to increase arginine abundance in the culture supernatants relative to those negative ones that harbored GTC (the SNP variations in the abovementioned allele of the 10 strains were validated by PCR amplification; Fig. 6A). Having validated this phenotypic difference, we introduced 6 out of these 10 strains [three positive strains with higher ability to increase arginine level in vitro: O1, O2, and O3; and three negative strains with lower ability to increase arginine level: Y1, Y2, and Y3] in a mouse model of d-galactose-induced aging and evaluated various behaviors and antioxidative parameters in the host mice after 9 weeks (Fig. 6B and C).

Our behavioral tests (Fig. 6D-I) indicated that aging significantly reduced the activity levels of mice in terms of the numbers of crossings and rearings in an open-field test. Aging also appeared to damage their learning and memory capacities, as indicated by marked decreases in spontaneous alterations during the Y maze test and an increase in the time spent in the target quadrant after training and a reduction in escape latency during a 5-day training period for the Morris water maze. Aging-related behavioral damage was reversed by both arginine supplementation and *B. longum* strain gavage (Fig. 6D-I). More significant alleviation of the behavioral effects was observed when positive strains (O1, O2, and O3 with the AGT allele) or arginine was administered. Specifically, the administration of strain O1, O2, O3, or arginine led to the significant recovery of five, four, four, and four of the five aging-related parameters, respectively, whereas the administration of negative strains (Y1, Y2, and Y3 with the GTC allele) had little effect on most parameters.

**Fig. 7:**
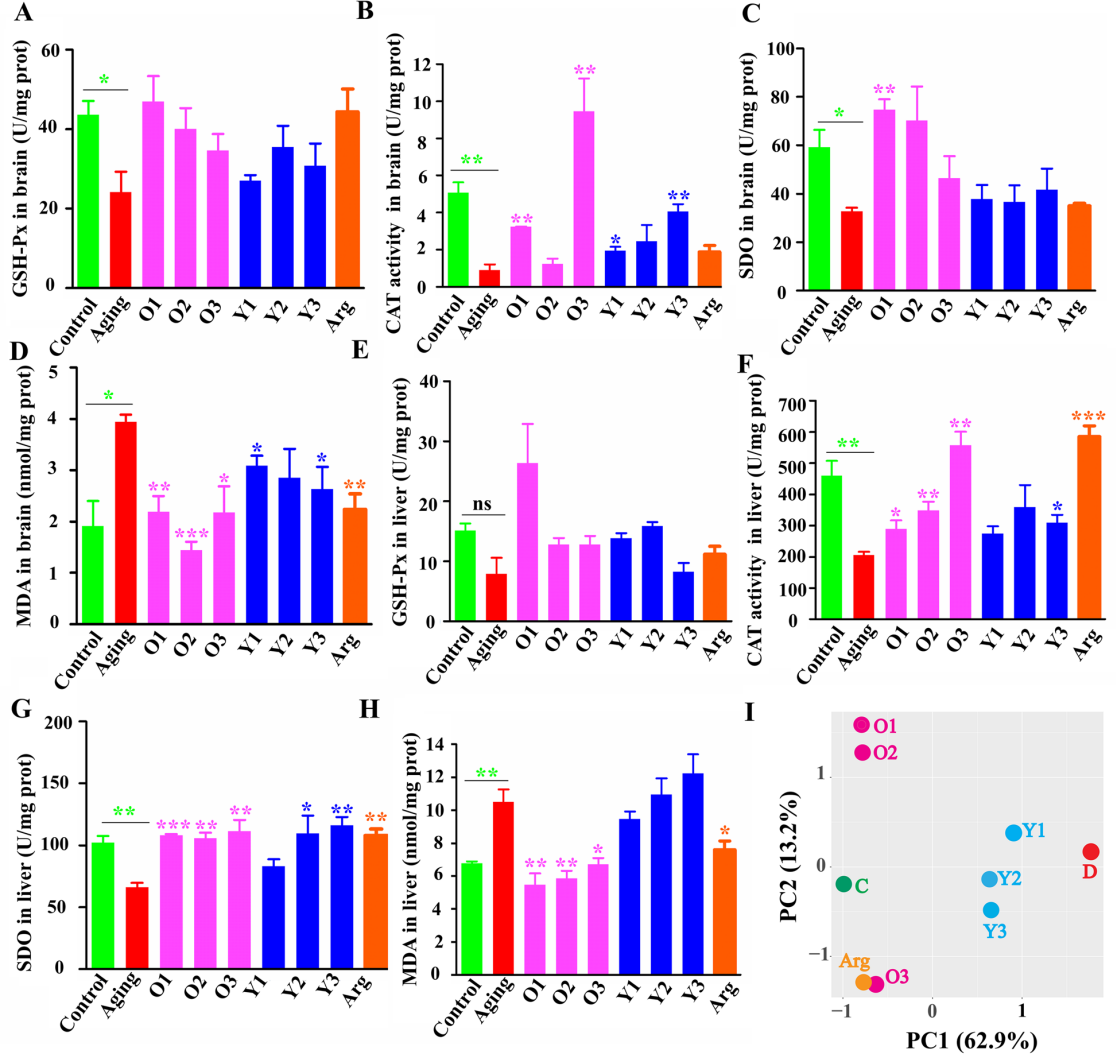
*B. longum* strains with specific genotypes can significantly reverse oxidative damage in aging mice. Oxidative parameters in brain tissue (A-D). Oxidative parameters in liver tissue (E-H). PCA plot based on all behavioral and oxidative data as the inputs (I). The normal distribution of all data was confirmed using the Kolmogorov–Smirnov (KS) normality test. All data were analyzed using one-way ANOVA and are presented as the means ± standard errors of the means; *n* ≥ 3 for each group. Arg, Arginine group. Statistical significance was calculated for the comparisons between the aging group and the control group (*s in green color), and for the comparisons between the aging group and each of strain treatment groups (*s in the other colors). *P < 0.05, **P < 0.01, ***P < 0.001

Regarding antioxidative parameters, seven of eight oxidative parameters in the brain and liver were markedly damaged; only glutathione peroxidase (GSH-Px) activity in the liver was spared (Fig. 7A-H). Although positive and negative strains had similar effects on catalase (CAT) activity in the brain and liver, the malondialdehyde (MDA) level in the brain, and superoxide dismutase (SOD) activity in the liver, these effects were more strongly induced by the positive strains. Positive strains could also rescue SOD activity in the brain and MDA levels in the liver, whereas negative strains had no observable effects.

PCA based on these tested behavioral and oxidative parameters indicated that the groups treated with positive strains or arginine were located more closely to the control group and were more clearly separated from the aging group than the negative strain groups. The data support our conclusions from the individual index and demonstrate that positive, rather than negative, *B. longum* strains can more efficiently alleviate aging (Fig. 7I). In addition, both *groEL* bifidobacterial profiling and quantitative PCR analysis consistently demonstrated that each of the six ingested *B. longum* strains engrafted successfully with colonized biomass of 10^8^–10^9^ cells/g feces at the time points of week 1, week 4, and week 9, thus suggesting their stable occurrence during intervention (Figure S15).

### The metabolic profile of gut microbiota was differentially modified according to the genotypes of administered *B. longum* strains

**Fig. 8:**
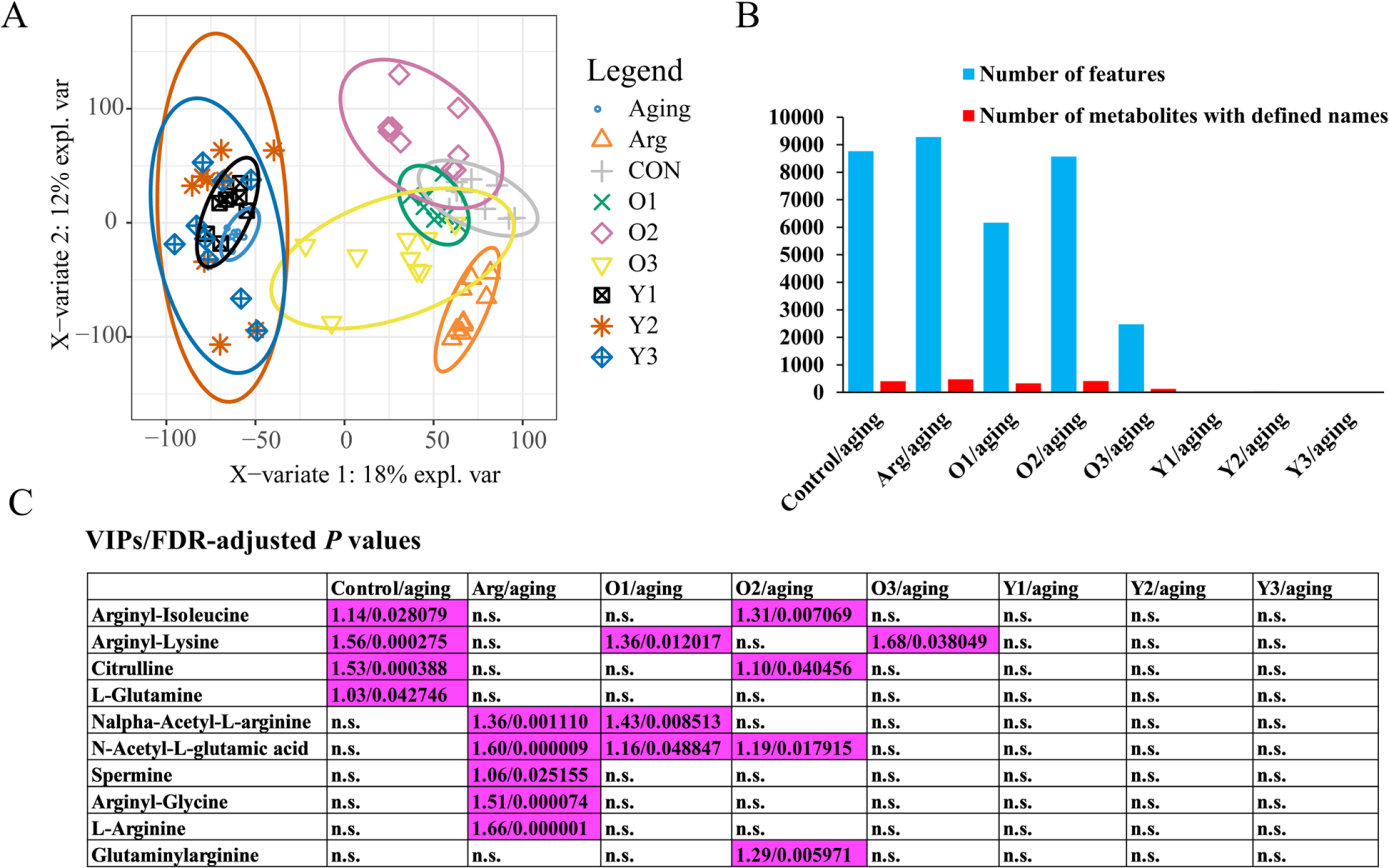
Aging-related metabolic profile in the gut microbiota was significantly reversed by the administration of arginine-enriching *B. longum* strains. **A** PLS-DA plots of fecal metabolite profiles according to the experimental group. **B** Numbers of metabolite features and metabolites with defined names, and trends in different concentrations between pairwise comparisons (FDR-adjusted *P* < 0.05 and VIP > 1). **C** Detected changes in metabolites related to arginine metabolism between pairwise comparisons. VIP values (before slash) and FDR-adjusted *P* values (after slash) are shown and highlighted in purple for all the identified differentially presented metabolites related to arginine metabolism between pairwise comparisons (FDR-adjusted *P* < 0.05 and VIP > 1). The cells marked by “n.s.” indicate that alterations of specific metabolites in specific comparisons did not reach significant level (FDR-adjusted *P* > 0.05 or VIP < 1). O series/Y series respectively denote treatments with individual positive strains/negative strains. Three positive strains are O1(278), O2 (RG4-1), and O3 (FJSWXJ10M2); three negative strains are Y1 (FGSZY16M3), Y2 (FHaNCM25GMM1), and Y3 (FSDLZ59M1). Arg, Arginine group

We also performed an analysis of differences in fecal metabolite profiles between treatments. A total of 22,074 fecal metabolite features remained after the data of nine experimental groups were processed. A PLS-DA based on these features indicated that both positive strains (O1, O2, and O3) and arginine played an important role in reversing age-related alterations in metabolites (Fig. 8A). These groups were clearly separated and distant from the aging group and had fecal metabolite patterns more similar to the control group. By contrast, the negative strain groups (Y1, Y2, and Y3) were intermixed with the aging group.

Next, we analyzed the metabolites for which different concentration trends had been identified between pairwise groups (FDR-adjusted *P* < 0.05 and orthogonal PLS-DA (OPLS-DA) VIP > 1). The OPLS-DA models were checked by cross-validation with 200 permutations to avoid overfitting (Table S14). A total of 8765 metabolite features (406 with defined names) in the aging group exhibited differentiation from the control group (Fig. 8B and Table S15). Supplementation with arginine or positive strains shifted 2474–9280 of the metabolite features (129–477 with defined names) relative to the aging group, whereas only 0–33 (0–1 with a defined name) identified metabolite features could distinguish the metabolomes of the negative strain groups from those of the aging group (Fig. 8B, Tables S16, S17, S18, S19, S20 and S21). We then summarized the differentially present metabolites related to arginine metabolism (Fig. 8C). We were intrigued to observe differences in arginine metabolism-related substances between the control and aging groups, which suggested that aging could perturb arginine flux in the fecal metabolome. Furthermore, both arginine supplementation and administration of positive strains affected the abundances of some metabolites involved in arginine metabolism, whereas negative strains did not exert observable effects. These results strongly suggested that *B. longum* strains with specific arginine biosynthesis pathway-related genotypes can recover aging-related metabolome perturbations and modify the arginine metabolic flux in the gut.

To further reveal the underlying changes in function, we conducted a pathway enrichment analysis based on fecal metabolites with known KEGG IDs (FDR-adjusted corrected *P* < 0.05; Figure S16). Compared with the control group, the aging group demonstrated evident differentiation of the fecal metabolites involved in some key pathways, including linoleic acid metabolism, α-linolenic acid metabolism, phenylalanine metabolism, and the PPAR signaling pathway. Supplementation with arginine or positive strains markedly modulated some of these aging-perturbed pathways, including linoleic acid metabolism, α-linolenic acid metabolism, and the PPAR signaling pathway. Additionally, both supplementation with arginine and the administration of O1 regulated tryptophan metabolism, whereas O2 modified the neurotrophin signaling pathway; this might be relevant to the alleviation of aging-related negative behavioral changes. Interestingly, linoleic acid metabolism was a core pathway affected by aging that was significantly modified by effective treatments (positive strains O1, O2, and O3).

Overall, these results indicate that *B. longum* strains with specific genotypes and superior arginine enrichment ability in vitro can recover aging-related perturbations in fecal metabolite profiles and modify the arginine flux in the gut. These strains could also affect other crucial biological pathways in the gut microbiota, such as linoleic acid metabolism.

## Discussion

In this study of the evolution, transmission, and associations of a gut microbial species with host phenotypes, we selected *B. longum* as a model species because it is a core colonizing species in the human gut microbiome, and its relative abundance is related to host age. Using a conceptual framework based on evolution and the pathogen transmission theory, we showed that *B. longum* had formed at least three geographically related populations and established the active transmission of *B. longum* strains across different types of hosts and according to geography and proximity. Interestingly, we identified a strong and statistically significant association between host age and genetic variations in *B. longum* genomes. We further correlated host metabolic flux with gut bacterial metabolic activity and provided an example to support the potential therapeutic application of this knowledge.

This study provided additional evidence that environmental conditions are related to the composition of the gut microbiota and the overall genotypic profile of an individual gut resident species (*B. longum*). Although geography is the primary discriminant, host age is also a significant contributing factor. Our findings support an earlier report by Zhang et al. that found that gut genera were clustered mainly according to host ethnicity/geography rather than lifestyle in Chinese cohorts [65]. Similarly, He et al*.* identified an isolated district as the top host factor that showed a significant association with gut microbial variations among the samples collected within Guangdong province [66]. Furthermore, in this study, we revealed that species-level composition profiles within the genus *Bifidobacterium* are largely discriminated by geography. Although previous research has focused on genetic and functional reservoirs in the human microbiome and their relationships with environmental factors [67–69], the effects of these factors on the overall genotypes of individual gut microbial species remained largely unknown. We determined that geographical location (country/province) contributed most to genetic variations in *B. longum*, followed by host age. The geographical dissimilarities at both the microbiome and genotype levels might be explained by the decreased transmission of gut microbes as the geographic scale increased, which was due to diminished environmental survival during fecal–oral transmission and limited human mobility.

Our data provide several insights into the evolution of *B. longum*, a gut resident microbe. Studies of strain-level evolution of pathogens and gut allochthonous bacteria have received considerable attention in recent years [18–20, 22] and have revealed valuable basic knowledge, as well as useful procedural methods. Here, we applied the theory and approach used in research of pathogens and gut allochthonous bacteria to the representative gut microbe *B. longum*. In this context, we observed the existence of distinct geographical populations of *B. longum* strains and identified population-specific microbial functional potential. First, multiple previous reports supported the existence of geographically based distributions of different strain patterns [22, 23, 72], which are usually named as “isolation by distance” brought about by host-microbe co-dispersal, possibly due to migration movements of early humans. Our findings suggested that despite increases in globalization and a multinational probiotic industry, the transmission bottleneck caused by geographical segregation across counties has inhibited genetic coalescence across distinct bacterial populations, and strengthened vertical transmission and seeding from the local (social) environment, resulting in geographically specific gene pools.

However, isolation by distance is likely not the only force acting on the genetics of *B. longum*; other metadata (e.g., different lifestyles, diet, and host genetics) might also have their influence. For example, significant SNP variations in cell wall biosynthesis-related genes and biofilm formation pathway in the core genomes of different *B. longum* populations might be a reflection of different degree of antibiotic exposure among subjects from different countries. Antibiotic stress has been reported to be able to induce bacterial biofilm formation [73] and alter expression of their cell wall biosynthesis-related genes [74]. Meanwhile, it has been confirmed that deletion of bacterial genes involved in cell wall biosynthesis [75] or overexpression bacterial genes that influence biofilm formation [76] led to significant changes of bacterial resistance against specific antibiotics. In addition, the carbohydrate metabolism-related gene profiles of strains from different populations were also markedly different, especially with respect to genes related to lactose and arabinose metabolism. Arabinose is a plant-derived polysaccharide that is enriched in high-fiber food that cannot be digested in the upper gut and is exclusively used by gut microbes. This observation supports the existence of selection pressure by different dietary habits on the genotypes of the strains [77], in which Chinese populations can be characterized by a pattern associated with a high-fiber diet. In a recent study, De Filippis et al. demonstrated that diet may select distinctive *Prevotella copri* strains with distinguishable functions, with weakened genetic potential for complex carbohydrate utilization and enhanced drug metabolism of strains during dietary ‘‘Westernization’’ (from high-fiber diet to high-protein and fat diet) [77]. Besides these genes involved in utilization of dietary carbohydrates, other genes in pathways of thiamine metabolism and metabolism of amino acids also varied between *B. longum* populations, possibly separately reflecting diet differences on vitamins and proteins. Notably, vitamin B1 is present in a wide range of food products, especially cereal bran, and it is heavily reduced in refined cereals, which are typically consumed in Western countries. Potentially, our approach to determine the evolutionary history and functional segregation of *B. longum* could be expanded to other bifidobacterial species, including gut allochthonous species.

Our data also suggest that the transmission of gut-colonizing *B. longum* is frequent. Unlike pathogens, commensal intestinal bacteria could be transmitted between humans to promote health by establishing, maintaining, and replenishing microbial diversity in the host gut microbiota. However, the manner by which commensal bacteria are transmitted remains unappreciated and poorly understood, despite the likely similarities between both. We frequently observed *B. longum* strains with the same genotypes among individuals within China, particularly in the guts of genetically unrelated subjects within the same community (e.g., Wuxi home for the elderly) and family members. This observation suggests that cohabitation or proximity drives transmission and that similar lifestyle and dietary habits exert uniform selection for colonization by specific *B. longum* strains. Active transmission may be partly dependent on the environmental survival of the strains. *B. longum* strains are relatively aerotolerant, and live isolates have been detected in environmental samples (e.g., soil and water). Therefore, transmission might be a general feature of multiple gut *Bifidobacterium* species. Moreover, the strains isolated from the same host in our study were derivatives of a common ancestor, consistent with a single colonization event as described previously for other gut commensal [78, 79] and pathogenic species [80].

Our data also provide a molecular basis for host–microbe coevolution, and this knowledge could feasibly be used to promote host health. The causal link between the gut microbiota and host aging has been investigated extensively, and microbiome-based therapies such as dietary interventions, probiotics, and fecal microbiota transplantation have been shown to efficiently alleviate host aging [81]. Some bacteria have been associated with a long human lifespan by analyzing the gut microbiota of centenarians, including *Faecalibacterium prausnitzii* [82], *Eubacterium limosum* [82], and particular health-associated groups (e.g., *Akkermansia*, *Bifidobacterium*, and *Christensenellaceae*) [34]. No chronological threshold or age is associated with an abrupt change in the microbiota composition; rather, these changes proceed gradually over time [83]. Here, we compared relative abundances of different gut bacterial types among host age segments, instead of solely focusing on the unique gut microbiota features of centenarians. We identified a strong negative association of the genus *Bifidobacterium* with host age, consistent with previous observations of reduced bifidobacterial counts in the elderly compared with the gut microbiota of two or three other age groups [84–87]. We further investigated the bifidobacterial species-level composition and identified *B. longum* as the most dominant of the core bifidobacterial species in the studied cohort. We further determined that the relative abundance of *B. longum* was also significantly correlated with host age. Interestingly, efforts to associate the genotype of this aging-related species with host age revealed a robustly significant association with the bacterial arginine biosynthesis pathway. The relevance of this association was further validated by the differential abilities of *B. longum* strains with different SNP variations in related genes to enrich arginine levels in vitro and the divergent abilities of these strains to alleviate host aging in vivo. Preliminarily, we attribute these effects to the abilities of positive *B. longum* strains with arginine metabolism-active genotypes to efficiently modulate the arginine flux and the overall gut microbiota metabolome.

Previous studies have demonstrated many molecular mechanisms by which microbiota may favorably affect host health and aging, based on principles designed to seek possible solutions to those changes experienced during the aging process, including (1) decreased immune system functioning (i.e., immunosenescence) and low-grade chronic inflammation (i.e., inflammaging); (2) inappropriate oxidative stress; (3) impaired gut barrier function; (4) decreased energy supply for colon epithelial cells; and (5) perturbed gut metabolism (e.g., lipid metabolism, glucose homeostasis, vitamin B and conjugated linoleic acid production), as reviewed by Vaiserman et al. [81]. Here, we propose another potent mechanistic route that key players (*B. longum*) in the gut microbiota are capable of generating age-related genomic adaptations in the arginine metabolism pathway, enhancing the bacterial arginine-enriching ability, further modifying arginine flux and the overall metabolome in the gut microbiota, and ultimately achieving protection against host aging. It should be mentioned that we also observed that negative strains with relatively lower arginine enrichment ability also showed alleviation on a few limited aging-related parameters. The possible explanations for this might be that negative strains worked through other reported mechanistic routes. In addition, d-galactose-induced aging mouse model used here has been reported to have some limitations. It was believed that this accelerated aging model could only mimic “natural aging,” but could not completely capture all phenotypes of aging [88–90]. In addition, there is evidence that there were strain differences of the mouse species with respect to aging-related phenotypes [91, 92]. However, d-galactose-induced aging mouse model has priority and is one of the most preferred models for the study of aging and age-related diseases because of the favorable outcome in terms of increased aging markers, its convenience, the least side effects, and the higher survival rate throughout the experimental period [93–96]. In our following studies, we will use natural aging mice of different genetic backgrounds (not only C57/BL6 used here) to further validate our conclusions.

## Conclusions

This study demonstrated the evolutionary pattern of a gut autochthonous bacterial species and identified certain gene elements associated with the host phenotypes. We have provided an early demonstration of the mechanisms by which host–microbial interactions (e.g., probiotic effector molecules or pathways) can be identified, based on associations between the host phenotypes and bacterial genotypes. Our findings support the concept of coevolution between gut microbes and the host, in which the host exerts selective pressure on the microbial genotypes, while benefitting from microbial genomic adaptation. Furthermore, geography-specific gene pools of gut species reinforce the potential localized use of probiotics and live biotherapeutics to increase their beneficial value. It remains unclear whether strains would exhibit better probiotic functionality when administered to individuals in the same regions where the strains were naturally found and isolated. By revealing the modes of active transmission, we demonstrate that the gut microbiota is an open reservoir that can be established, maintained, and replenished, and this provides a basis for microbiome-targeted therapeutics.

## Supplementary Information

**Additional file 1.** Supplementary materials.

**Additional file 2.** Supplementary tables.

## Data Availability

The *16S rRNA* gene sequencing data and *Bifidobacterium* composition data were submitted to the Sequence Read Archive (SRA) under BioProjects PRJNA665348 and PRJNA665364, respectively. The genome data of the 143 newly sequenced strains were submitted to the SRA under BioProject PRJNA665750.

## Abbreviations

GWAS: Genome-wide association studies
tb-RDA: Transformation‐based redundancy analysis
db-RDA: Distance-based redundancy analysis
DCA: Detrended correspondence analysis
PERMANOVA: Permutational multivariate analysis of variance
SNP: Single nucleotide polymorphism
COG: Cluster of orthologous group
KEGG: Kyoto Encyclopedia of Genes and Genomes
SCG: Semi-clonal groups
CGs: Clonal groups
MDS: Multidimensional scaling
MWM: Morris water maze
GSH-Px: Glutathione peroxidase
MDA: Malondialdehyde
CAT: Catalase
SOD: Superoxide dismutase
PCA: Principal component analysis
PLS-DA: Partial least-squares discriminant analysis
VIP: Variable important in projection
FDR: False discovery rate
