# Supplementary material for: Human gut-derived B. longum subsp. longum strains protect against aging in a d-galactose-induced aging mouse model
Source: Microbiome. 2021 Sep 1;9:180. doi: 10.1186/s40168-021-01108-8 (PMC8411540; doi:10.1186/s40168-021-01108-8)
Supplement: Supplementary file 2 — Additional file 1. Supplementary materials. [file 40168_2021_1108_MOESM2_ESM.docx]

**Supplementary Methods**

**Open-field test**

The open-field test was conducted as previously described[[1-3](#_ENREF_1)]. In brief, the tests were performed on an open arena (50×50×50 cm, with the floor divided into 25 equal squares) with a single 40-W bulb (3000 lux). Each mouse was released in the middle of the chamber, and left for 1 min for environment adaptation. Two observers separately recorded the activity of each individual mouse (including crossings, rearings, leanings, and washings) for 5 min. Data on horizontal (crossings) and vertical (rearings) activity were used for the analysis.

**Morris water maze (MWM) test**

The MWM test was conducted as described previously[[1-3](#_ENREF_1)]. Briefly, tests were performed in equipment consisted of a circular water tank (120 cm in diameter, 35 cm in height) filled with 22 °C–24 °C water to a depth of 15.5 cm, which was rendered opaque by adding ink. A platform (4.5 cm in diameter, 14.5 cm in height) was placed at the midpoint of one quadrant and hidden 1–1.5 cm below the surface of the water. In a training experiment, each individual mouse received four training trials per day for 5 consecutive days to find the hidden platform within 60 s. Escape latency (time spent finding the platform) was calculated for each trial. On day 6, the platform was removed and the probe test was carried out. The time that mice spent swimming in the former platform-containing quadrant (target quadrant) was measured. All data were automatically recorded by a computerized video system.

**Step-through test and Y-maze test**

The step-through test was performed according to a previous report[[4](#_ENREF_4)]. The Y-maze test was conducted as previously described[[5](#_ENREF_5)].

**Occurrence of ingested *B. longum* strains**

Fecal samples of mice in nine experimental groups collected at time points of baseline, the week 1, the week 4 and the week 9 were used. Samples from three mice were randomly selected for analysis of each treatment at each time point. Two approaches were independently used to access the presence of each of administrated *B. longum* strains in the gut of mice. Fecal DNA was extracted by using FastDNA Spin Kit for Soil (Catalog number: 116570200, MP Biomedicals, USA) with additional mechanical and enzymatic cell-lysis steps as described in the methods section under subtitle “Microbiota analysis”. The bifidobacterial composition analysis from wet experiments to bioinformatics analysis was based on the 60 kDa chaperonin (*groEL*) gene amplicon profiling and performed as described in the methods section under subtitle “Microbiota analysis”.

For quantitative real-time PCR (qPCR), qPCR was performed using a Bio-Rad CFX96TM Real-Time System. Each of the six *B. longum* strains was respectively quantified with species-specific primers (F: 5-TTCCAGTTGATCGCATGGTCTTCT-3; R: 5- GGCTACCCGTCGAAGCCACG-3)[[6](#_ENREF_6), [7](#_ENREF_7)]. Positive control with genomic DNA of each of the target *B. longum* strains as template and negative control using water instead of genomic DNA were included in all PCR runs. The PCR system (20μL) consisted of 2μL fecal genomic DNA (template), 10μL 2 × Bio-Rad iTaqTM Universal SYBR® Green Supermix, 2 µl forward primer (0.4 µmol in the final mixture), 2 µl reverse primer (0.4 µmol in the final mixture), and 4 µl ddH_2_O. The following qPCR program was finally used: an initial denaturation step at 95°C for 2 min; 35 cycles of denaturation at 95°C for 5 s and annealing/extension at 60°C for 30 s; a melt curve analysis between 65°C and 95°C in 0.5°C increments at 2–5 s/step; and polymerase activation and DNA denaturation at 95°C for 5 min. For absolute quantification, feces-based standard curves were prepared. In brief, 1 ml overnight liquid cultures (16 h) of each of the *B. longum* strains (for which cell numbers were determined by quantitative culture) together with 0.1g baseline feces (without each of the target *B. longum* strains) were pooled, washed twice by sterile phosphate saline (PBS, pH 7.0) and then under DNA extraction to yield initial stock DNA for each strain separately. A ten-fold dilution series of the above-mentioned stock DNA was used for preparing a standard curve for each target *B. longum* strain. The detection limit of the qPCR system for each strain was determined during making the corresponding standard curve.

**Metabolomics of the gut microbiota**

A fecal sample from each individual mouse (50 mg, n=9 for each treatment) was collected in a 2 ml tube at the time of day 64. Samples were immediately quenched in liquid nitrogen and stored at −80 °C. A steel ball (6 mm in diameter), 400 μL of a pre-cooled mixture of methanol and ultra-pure water (4:1, v/v), and 20 µL of an internal standard (0.3 mg/mL L-2-chloro-phenylalanine in acetonitrile) were added to each tube. The fecal samples were then grinded by a frozen tissue grinder (−10 °C, 50 Hz), followed by ultrasonic extraction for 30 min (5 °C, 40 KHz). After ultrasonication, samples were kept at −20 °C for 30 min. The samples were centrifuged (13000 g, 4°C) and the resulting supernatant was prepared for liquid chromatograph–mass spectrometry (LC-MS) analysis. A quality control (QC) sample was prepared by pooling aliquots of all the samples.

The UHPLC-Q Exactive system (Thermo Fisher) with a BEH C18 column (100×2.1 mm i.d., 1.7 µm; Waters, Milford, USA) was used for LC-MS analysis. The parameters of LC-MS were as follows: for mobile phase A, 0.1% formic acid in water, and for mobile phase B, 0.1% formic acid in acetonitrile/isopropanol (1:1, v/v). The solvent gradient was as follows: t = 0 min, 5% B; t = 3 min, 20% B; t = 9 min, 95% B; t = 13 min, 95% B; t = 13.1 min, 5% B; and t = 16 min, 5% B. The flow rate was 0.4 ml/min, the injection volume was 0.2 μL, and the column temperature was maintained at 40 °C. Mass spectrometry analysis was conducted in both the positive ion mode and the negative ion mode, and key parameters were as follows: m/z, 70–1050; sheath gas flow rate, 40 psi; aus gas flow rate, 10 psi; aus gas heater temp, 400 °C; ionSpray voltage floating (ESI+), +3500 V; ionSpray voltage floating (ESI˗), −2800 V; and normalized collision energy, 20-40-60 V. The QC sample was injected into the LC-MS system every 10 analyzed samples to observe and correct the repeatability of the analysis.

For data processing, raw data were under baseline filtering, peak recognition, integration, retention time correction and peak alignment in the software Progenesis QI (Waters Corporation, Milford, USA), and the resulting data matrix possessed a retention time, m/z, and peak area. The data matrix was further processed as follows: 1) retained features that were present in at least 80% of samples within each experimental group; 2) used minimal value in raw data to fill the missing value; 3) conducted data normalization by the peak area integration method, and deleted features with a relative standard deviation (RSD) of more than 30% in QC samples; and 4) performed log transformation on the data. After data processing, the obtained features were identified in the METLIN (<https://metlin.scripps.edu/>), HMDB (<http://www.hmdb.ca/>), and self-constructed local databases.

**Supplementary Results**

**Population-specific genomic loci suggested significant variations in cell wall biosynthesis and carbohydrate metabolism among populations**

This analysis identified 724 SNPs in coding regions (215 non-synonymous, 508 synonymous, and one terminating mutation) and 362 genes that were specific to single populations (Tables S7 and S8). These population-specific variations were distributed widely across multiple phylogenetic branches of the corresponding populations (Figure S9), indicating that they were not formed solely by clonality in the population structure but might have been gained and/or lost independently via multiple events. Population-specific variations were annotated and categorized according to predicted gene functions (COG) and KEGG pathway terms for further biological explorations.

Regarding the SNPs in the core genome, 75% (543/724) and 47.4% (343/724) had functional matches in the COG and KEGG databases, respectively, and these accounted for 76.4% (553/724) of all population-specific loci (Table S7 and Figure 3A and 3B). Functional enrichment analyses indicated significantly higher frequencies of terms such as ‘cell wall/membrane/envelope biogenesis’, ‘peptidoglycan biosynthesis’, and ‘biofilm formation - *Pseudomonas aeruginosa*’ against the background of the expected distribution from a reference genome (Table S9 and S10 and Figure 3A and 3B; one-sided Fisher test, *P*=1.50×10^˗5^, *P*=8.84×10^˗14^, and *P*=5.27×10^˗8^, respectively). Notably, cell wall/membrane/envelope biogenesis was the only significantly enriched function among the 20 detected COGs (Table S9, Figure 3A), and this category encompassed 66 SNPs in 14 genes (non-synonymous mutations present in 10 genes), in which *murE* (BL1356), encoding a key enzyme for cell-wall peptidoglycan biosynthesis, contained the largest number of variations (seven non-synonymous sites among 13 SNPs). KEGG enrichment analysis further confirmed these results (Table S10 and Figure 3B) and indicated that peptidoglycan biosynthesis was the most significantly elevated pathway (*P*=8.84×10^˗14^), with 58 SNPs (18 non-synonymous) in nine genes. The biofilm formation pathway was also identified as important, with 21 SNPs (four non-synonymous) in two genes, compared with the detection of only five genes in this pathway in the reference genome NCC2705. Interestingly, one gene with a single putative lacto-N-biose phosphorylase C-terminal domain, *gnpA* (BL1641), was not assigned to any function or term but had the greatest number of variations (54 SNPs with nine non-synonymous sites). Further investigation of this gene is warranted. Overall, cell wall biosynthesis, particularly peptidoglycan biosynthesis, was the most significant discriminant among the three populations as evidenced by both KEGG and COG enrichment analyses. SNPs were accumulated densely in individual genes and frequently involved non-synonymous loci, and these alterations could well have direct effects on strain phenotypes such as gut fitness.

In the accessory genome, only the carbohydrate transport and metabolism category was identified as significant in COG analysis (Table S11 and Figure 4, one-sided Fisher test: *P*=1.39×10^˗5^), and no significant KEGG pathways were detected (Table S12). The carbohydrate transport and metabolism category involved 52 differentially present genes (Figure S10A), while genes that encode various enzymes involved in the transport and metabolism of arabinose (14/52, Figure S10B) and lactose (9/52, Figure S10C) exhibited the greatest differences. With respect to the arabinose metabolism-related genes, the BLAsia2 and BLOthers profiles were more similar and were distinct from that of BLAsia1 (Figure S10B). Compared with the other two populations, BLAsia1 exhibited relative enrichment of group_1555, whereas the other genes were largely absent. By contrast, group_4149 and *xsa* were more frequently detected in BLAsia2. Notably, these discriminant genes included two genes that encode paralogs: *araE* and *araE_1*, and *araN_1* and *araN_4* (Figure S11). We determined that none of the strains from BLAsia1 harbored *araN_4*, whereas the strains from BLAsia2 and BLOthers harbored both *araN_4* and *araN_1*. We also observed the significant enrichment of *araN* (i.e., sum of the number of *araN_1*, *araN_2*, *araN_3*, *araN_4*, and *araN_5*) in BLAsia1 (Mann–Whitney test: *P*=0.003 for BLAsia1 versus BLAsia2, and *P*<0.001 for BLAsia1 versus BLothers). The distributions of *araE* and *araE_1* were also highly variable. The overall numbers of *araE* genes (*araE*, *araE_1*, and *araE_2*) did not differ significantly between BLAsia1 and the other two populations (Mann–Whitney test: *P*=0.351 for BLAsia1 versus BLAsia2, and *P*=0.057 for BLAsia1 versus BLOthers). However, an elevated number of *araE* genes was observed in BLothers relative to BLAsia2 (Mann–Whitney test: *P*=0.011). The sequences of these gene paralogs exhibited a high level of dissimilarity (Figure S11).

Among the genes involved in lactose metabolism (Figure S10C), group_3517 was identified as a core gene in BLAsia1 but was largely absent from BLAsia2 and BLOthers. By contrast, group_6062 was enriched in BLAsia2 but was present at relatively low frequencies in BLAsia1 (4/124) and BLOthers (5/74). The paralog compositions of *lacS*, *lacF*, and *lacZ* varied widely between the populations, and their gene numbers were also markedly distinct between the populations (Mann–Whitney test: *P*<0.05 for each paired comparison). The paralog sequences of these genes were highly dissimilar (Figure S12 and S13). Generally, genes involved in arabinose and lactose transport and metabolism varied markedly between the three populations in both the presence or absence of genes and the paralog compositions of specific genes.

***B. longum* undergoes active transmission within families and communities, via inter-city, and inter-country routes, and across different hosts**

A phylogeny-based approach could not be used for *B. longum* to capture the spread pattern at the species level because recombination confuses the vertical genetic signals and prevents an understanding of the true evolutionary history. In this study, we concentrated solely on recently formed clonal groups in which recombination was limited. We determined the recombination regions in each of the 31 semi-clonal groups (SCGs; pairwise SNP distance <2500 among strains within each SCG) using Gubbins, discarded the SNPs detected in recombined regions and re-calculated the SNP distances. Finally, we defined 46 clonal groups (CGs) in which the pair-wise SNP distance was less than 10 (Table S13). The strains within each CG were closely phylogenetically related and were most likely to be descendants of a common ancestor.

Several geographic levels of transmission have been identified. Inter-country spread was observed between strains from Korea and Italy (P29). The 1898B of P29 was isolated from the feces of a neonate in Italy[[8](#_ENREF_8)], and BG7 of P29 was isolated from a human subject in Korea. Three strains identified in commercial probiotic products in the United States (specifically, California and Maryland) and strain 35624, which was isolated from an adult intestine in Ireland, were also clonally related (CG8). However, the latter USA–Ireland transmission was not definite and might have resulted from the purchase and use of probiotic products. In China, active inter-provincial spread was identified between Jiangsu and Guangdong (CG3 and P6); between Shandong and Guangdong (CG4); between Beijing, Guangxi, and Jiangsu (CG5); between Sichuan and Guangdong (P3) and between Jiangsu and Anhui (P4), and Guangdong appears to serve as a hotspot in these patterns. Similarly, transmission between different cities within the same province (CG4) has also been observed. Overall, our analysis indicates the existence of inter-country, inter-provincial and inter-city transmission of *B. longum* strains, particularly in China, which suggests a possible association with population migration.

Interestingly, we also observed transmission between individuals in close proximity. An analysis of 16 strains from 16 residents of a home for the elderly in Wuxi, Jiangsu yielded five distinct clonal groups (CG6, CG11, P8, P9 and P26), which suggested strain transmission within the community. Transmission between family members has also been identified. Specifically, *B. longum* strains in CG12 were transmitted across three generations (65-year-old grandmother, 36-year-old mother, and 1.4-year-old child), whereas for strains of P15 and P31, mother–child transmission was also observed but could not be clearly classified as vertical transmission or transmission by proximity. Furthermore, we observed transmission of *B. longum* strains between a human and chicken (P10) in the same household.

The isolation of multiple strains within an individual sample was also of interest. We observed that strains isolated from the same individual were clonally related (CG1, CG2, P5, P10, P17, P21, P22, P23 and P32 for nine different human subjects) with a median pairwise SNP value of 2, consistent with a single colonisation event in which an individual subject was colonised by a unique clone.

Supplementary Table S4 Abundant genera identified in this study.

| Genus | Proportion in total used sequences (%) | Prevalence (%) | Median (%) | Range (%) |
| --- | --- | --- | --- | --- |
| *Bacteroides* | 16.55 | 100.0% | 12.84 | 0.096-58.66 |
| *Ruminococcaceae_unclassified* | 9.92 | 100.0% | 9.65 | 0.163-37.47 |
| *Enterobacteriaceae_unclassified* | 9.48 | 100.0% | 3.16 | 0.162-75.57 |
| *Prevotella* | 9.26 | 100.0% | 0.27 | 0.013-54.40 |
| *Lachnospiraceae_unclassified* | 6.83 | 100.0% | 5.79 | 0.138-23.81 |
| *Bifidobacterium* | 4.28 | 100.0% | 0.97 | 0.049-68.71 |
| *Lactobacillus* | 3.50 | 100.0% | 0.14 | 0.012-66.61 |
| *Blautia* | 2.44 | 100.0% | 1.73 | 0.032-12.62 |
| *Clostridiaceae_Other* | 2.41 | 100.0% | 0.71 | 0.018-28.92 |
| *Ruminococcus* | 2.09 | 100.0% | 0.75 | 0.029-21.92 |
| *Parabacteroides* | 2.04 | 100.0% | 1.10 | 0.009-14.50 |
| *Klebsiella* | 1.93 | 100.0% | 0.10 | 0.003-41.79 |
| *Megamonas* | 1.85 | 100.0% | 0.05 | 0.007-18.58 |
| *Streptococcus* | 1.38 | 100.0% | 0.24 | 0.015-20.03 |
| *Lachnospiraceae[Ruminococcus]* | 1.28 | 100.0% | 0.79 | 0.029-8.37 |
| *Collinsella* | 1.18 | 100.0% | 0.43 | 0.006-24.56 |
| *Dialister* | 1.09 | 100.0% | 0.04 | 0.005-27.37 |
| *Lachnospira* | 1.08 | 100.0% | 0.52 | 0.016-6.50 |

Only the genera with more than 1% of total sequences are listed.

Supplementary Table S6 Abundant *Bifidobacterium* species identified in this study.

| Species | Proportion in total used sequences (%) | Prevalence (%) | Median (%) | Range (%) |
| --- | --- | --- | --- | --- |
| *B. longum* | 36.08 | 100.0% | 29.79 | 0.79-94.50 |
| *B. pseudocatenulatum* | 22.05 | 100.0% | 6.97 | 0.66-89.85 |
| *B. adolescentis* | 14.61 | 100.0% | 1.41 | 0.52-81.24 |
| *B. bifidum* | 9.89 | 100.0% | 0.95 | 0.15-96.13 |
| *B. dentium* | 5.95 | 100.0% | 0.74 | 0.18-94.12 |
| *B. ruminantium* | 3.98 | 100.0% | 0.34 | 0.12-68.16 |
| *B. catenulatum* | 3.30 | 100.0% | 0.43 | 0.04-80.29 |

Supplementary Table S14 Model statistics for the multivariate analysis of the normalized metabolite concentrations obtained from LC-MS metabolomics^a^.

|  |  |  | **Model statistics** | | | | |
| --- | --- | --- | --- | --- | --- | --- | --- |
| **Model description** | **Type** | **Model type** | **R2X(cum)** | **R2Y(cum)** | **Q2(cum)** | **pR2Y** | **pQ2** |
| Control vs Aging | Supervised | OPLS-DA | 0.371 | 0.997 | 0.957 | 0.005 | 0.005 |
| Aging vs Arginine | Supervised | OPLS-DA | 0.412 | 0.999 | 0.973 | 0.005 | 0.005 |
| Aging vs O1 | Supervised | OPLS-DA | 0.406 | 0.996 | 0.961 | 0.005 | 0.005 |
| Aging vs O2 | Supervised | OPLS-DA | 0.402 | 0.996 | 0.958 | 0.005 | 0.005 |
| Aging vs O3 | Supervised | OPLS-DA | 0.295 | 0.976 | 0.804 | 0.005 | 0.005 |
| Aging vs Y1 | Supervised | OPLS-DA | 0.277 | 0.96 | 0.622 | 0.01 | 0.005 |
| Aging vs Y2 | Supervised | OPLS-DA | 0.24 | 0.966 | 0.635 | 0.005 | 0.005 |
| Aging vs Y3 | Supervised | OPLS-DA | 0.241 | 0.926 | 0.515 | 0.06 | 0.01 |

^a^cross-validation and testing was conducted with 200 permutations and 7 of cross-validation segments.


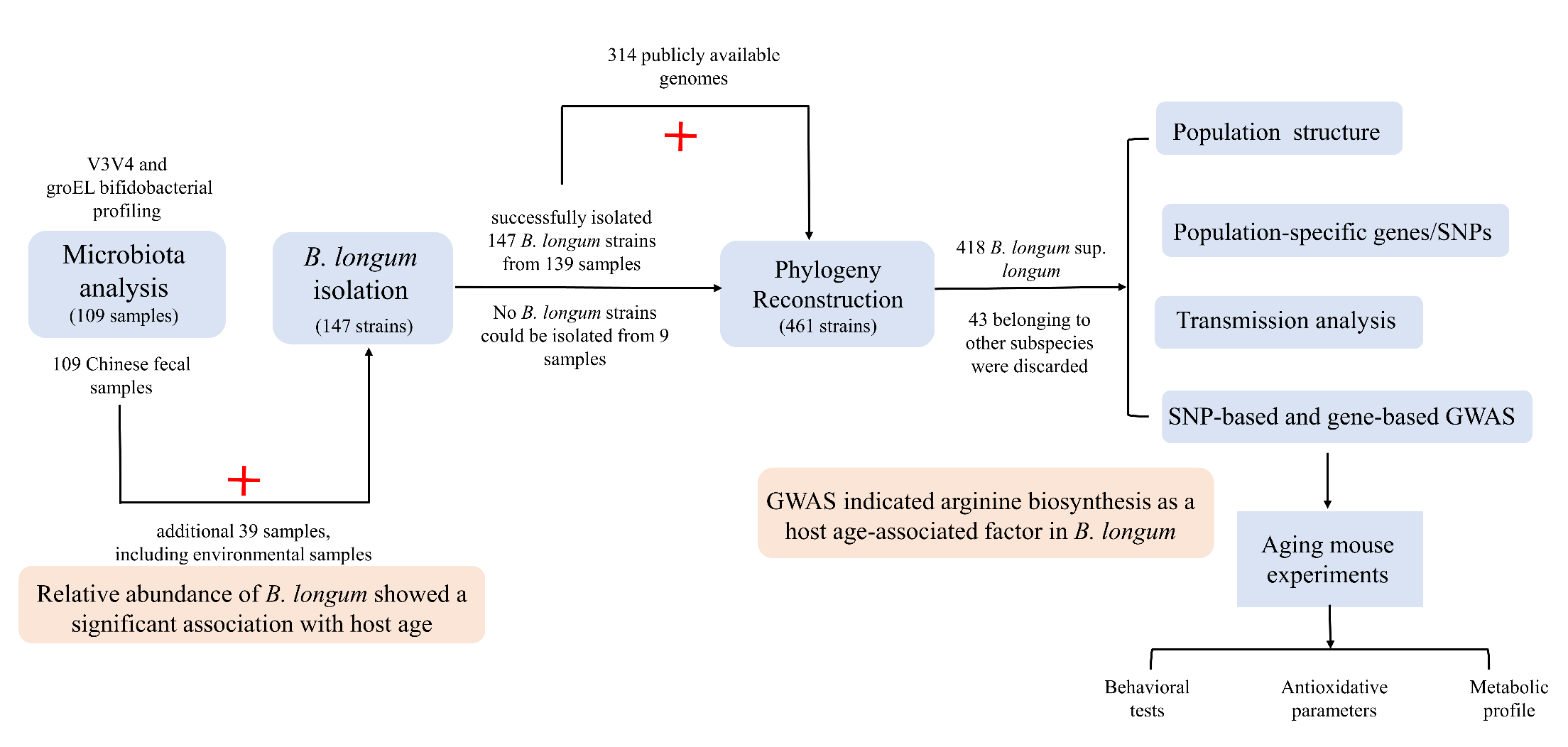


Figure S1. The flow chart of the approaches used for all the included analyses in this study. GWAS: Genome-wide association studies.


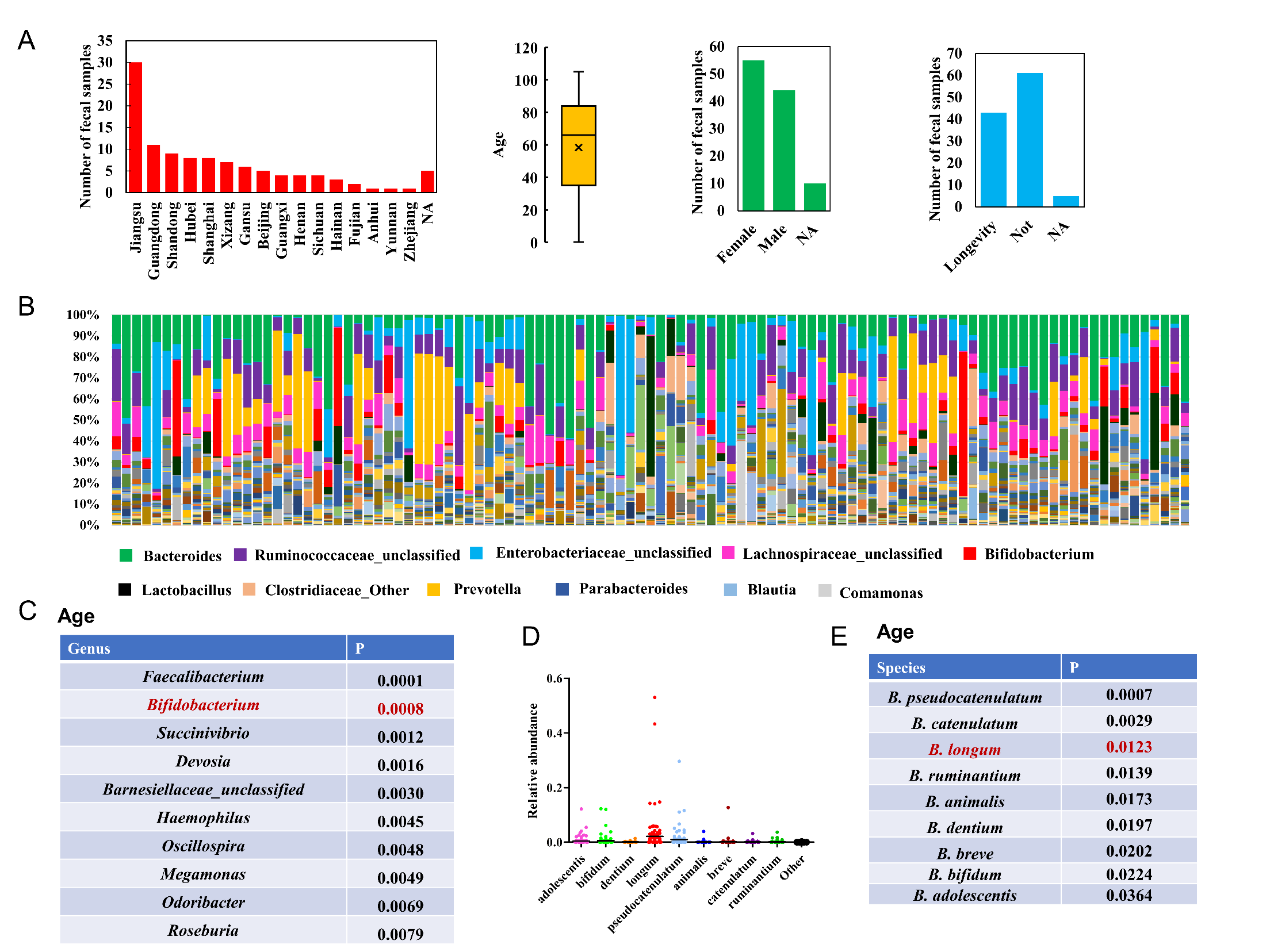


Figure S2. Background information (A), genus-level composition (B), comparisons of relative abundances of bacterial genera among host age categories (C), relative abundance of each bifidobacterial species (D) and comparisons of relative abundance of each bifidobacterial species among host age categories (E) based on the gut microbiota profile of a Chinese cohort. In panel C, the top 10 genera with significantly different relative abundances among age segments were listed. For the full list, please see Table S5. “Longevity” in panel A is equal to “longevous district status”.


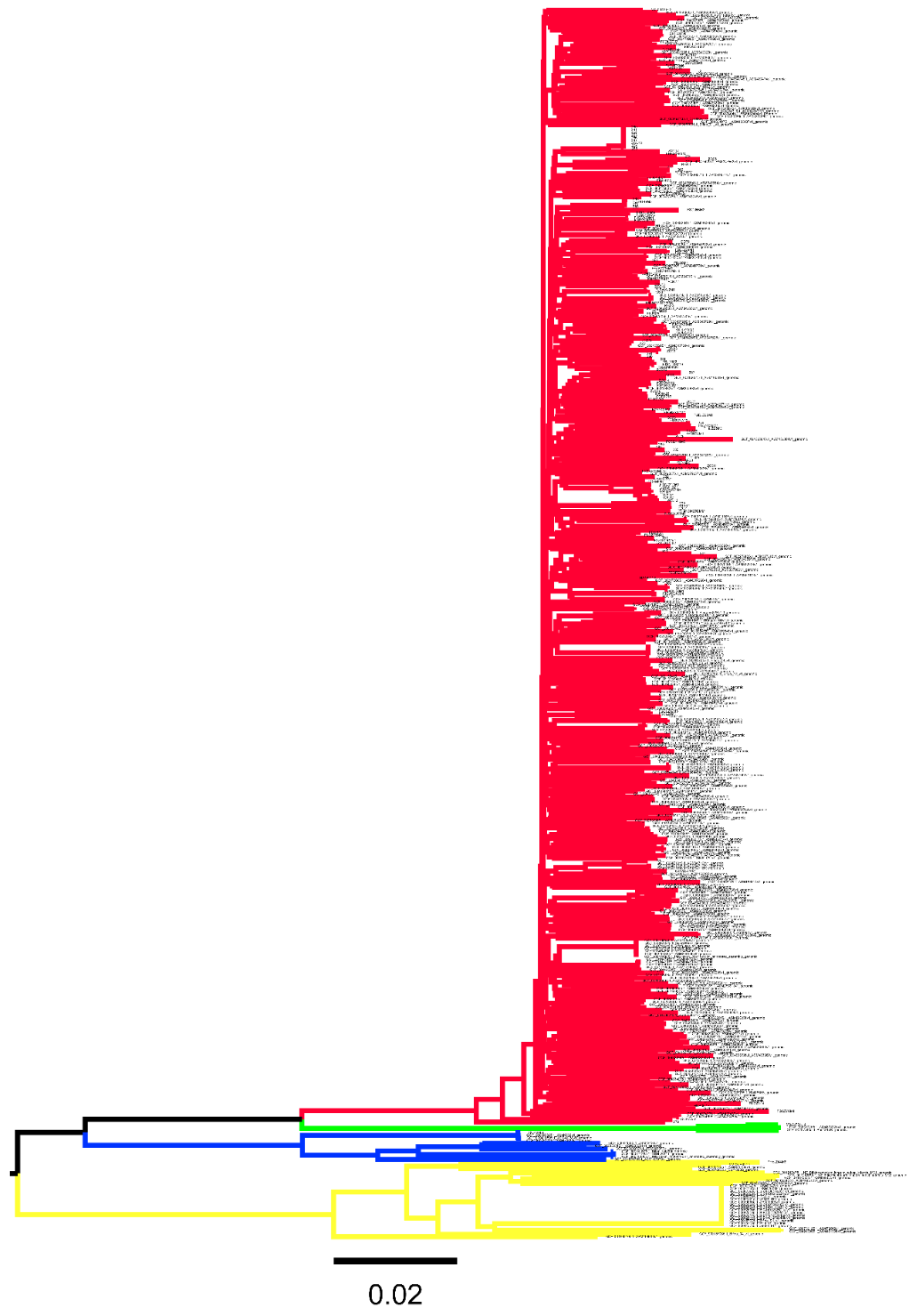


Figure S3. Neighbour-joining (NJ) tree of 461 *B. longum* strains based on core-genome bi-SNPs. Subspecies are indicated by different colors of corresponding clades, in which strains of *B. longum* subsp. *longum* are in red, strains of *B. longum* subsp. *suillum* are in green, strains of *B. longum* subsp. *infantis* are in yellow, and strains of *B. longum* subsp. *suis* are in blue.


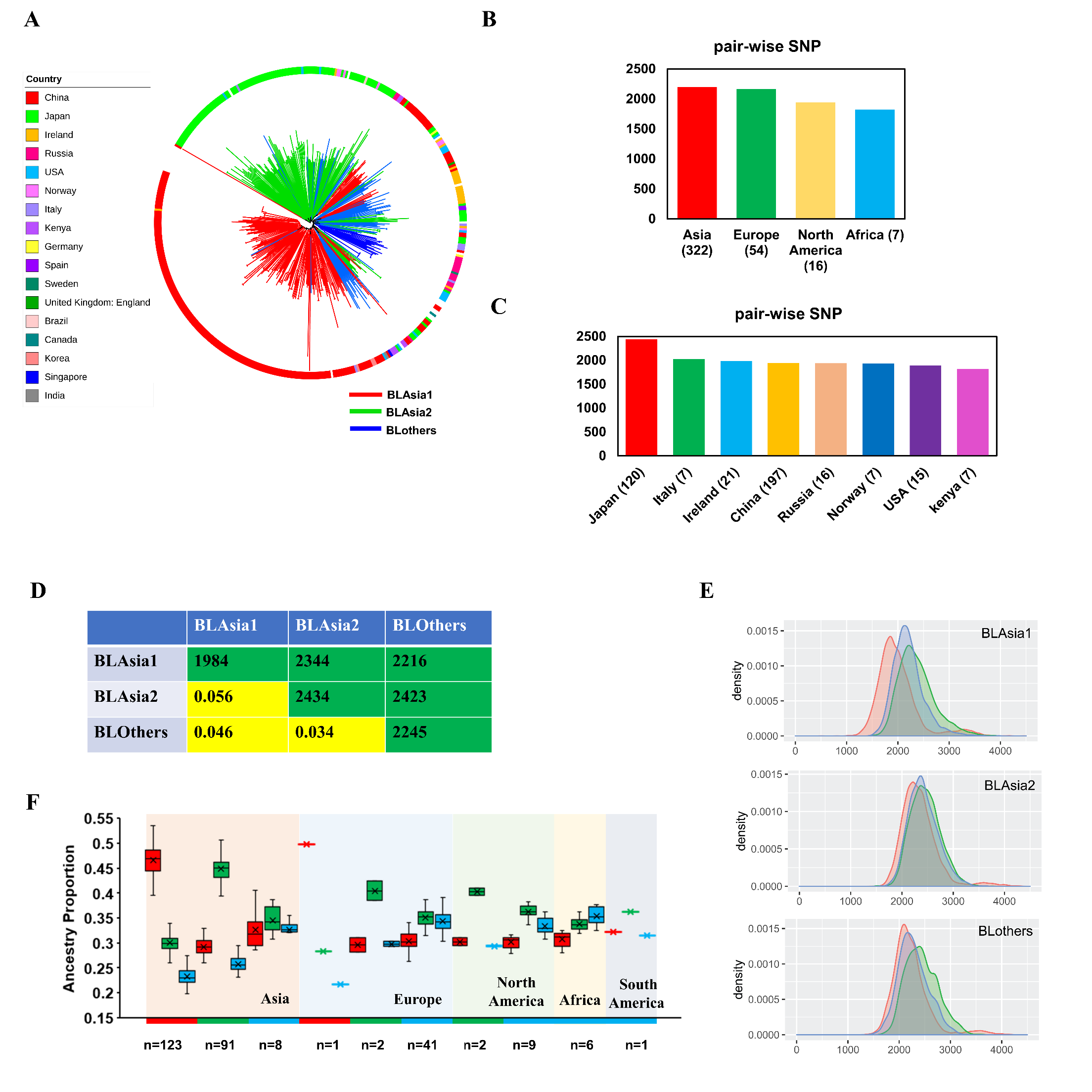


Figure S4. Population structure and divergence of *B. longum* and genomic diversity across geographical locations and between populations. (A) Neighbour-joining (NJ) tree of 418 *B. longum* strains based on 44752 SNPs. Populations revealed by fineSTRUCTURE are marked by different branch colors. The colors in the outer strip indicate the geographical locations where individual isolates were sampled. A missing value is represented by a blank. (B) Pairwise SNP distances among strains according to continent. (C) Pairwise SNP distances among strains according to country. (D) The degrees of divergence (Fst value, yellow background) and average SNP distances (green background) within and between populations, calculated based on 295 representative strains. (E) Distribution of SNP distances within and between populations, calculated based on 295 representative strains. Red, BLAsia1; green, BLAsia2; blue, BLothers. (F) *B. longum* ancestral distributions by geographical location. Strip colors at the bottom indicate *B. longum* populations and are consistent with the branch colors in panel A. The colors of blocks indicate different ancestry components of the three populations. Background colors indicate the respective continents as marked.


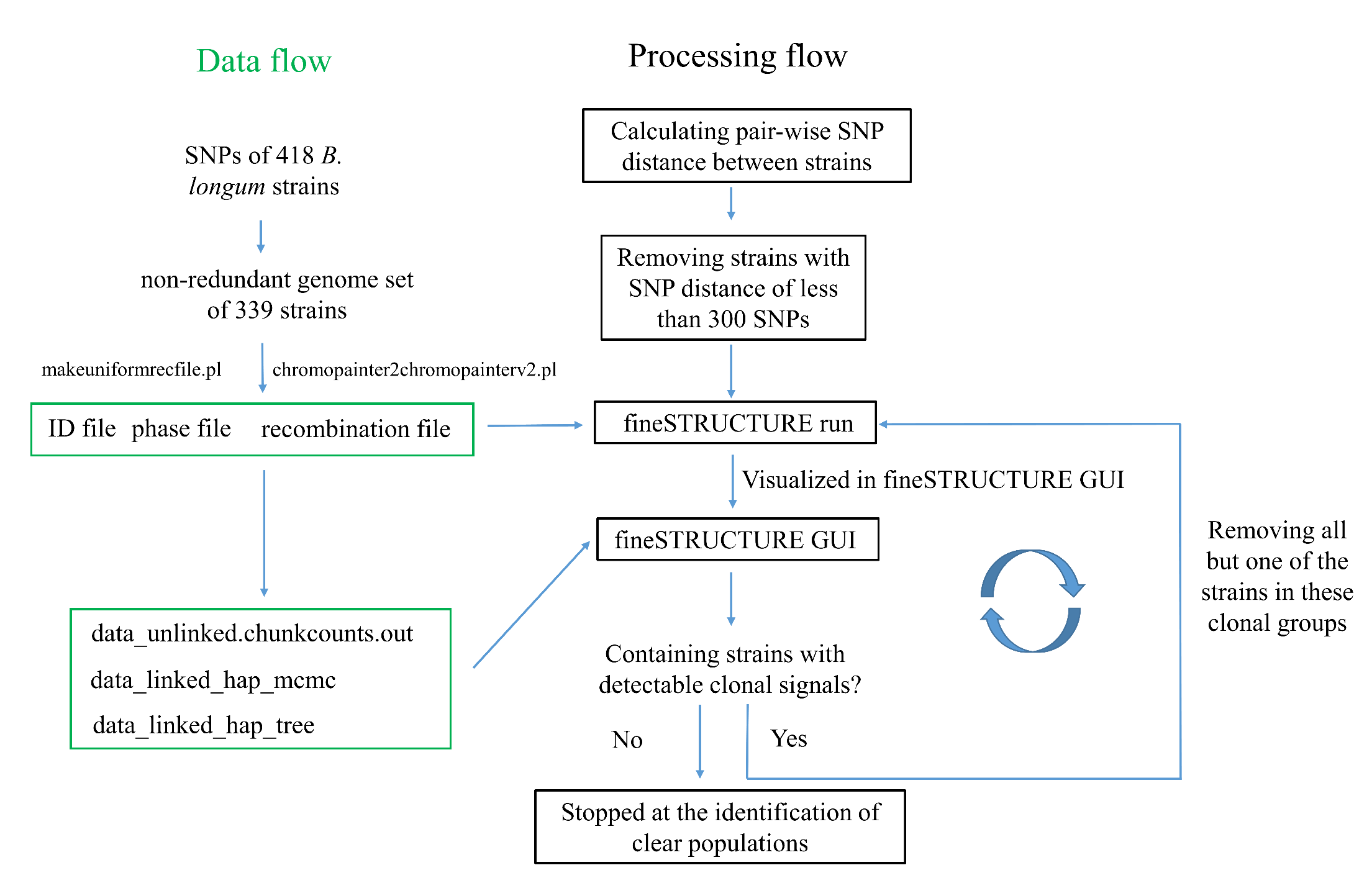


Figure S5. The flow chart of approaches used for population structure analysis.


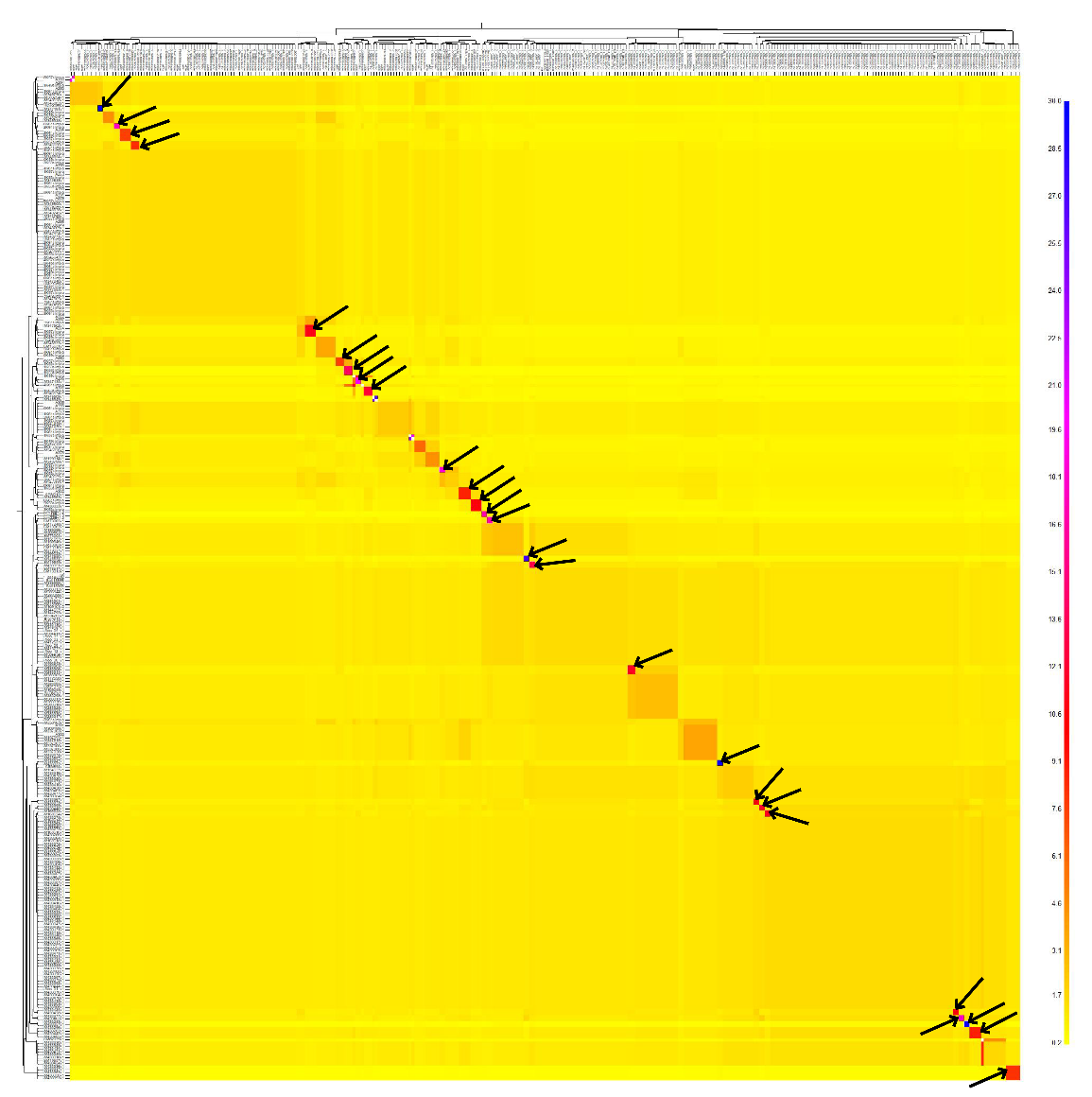


Figure S6. The co-ancestry matrix of *B. longum* strains after the first FineSTRUCTURE run. The color of each cell in the co-ancestry matrix represents the anticipated number of DNA chunks imported from a donor strain (column) to a recipient strain (row). The arrows indicate the detected clonal signal that should be removed in the following FineSTRUCTURE run.


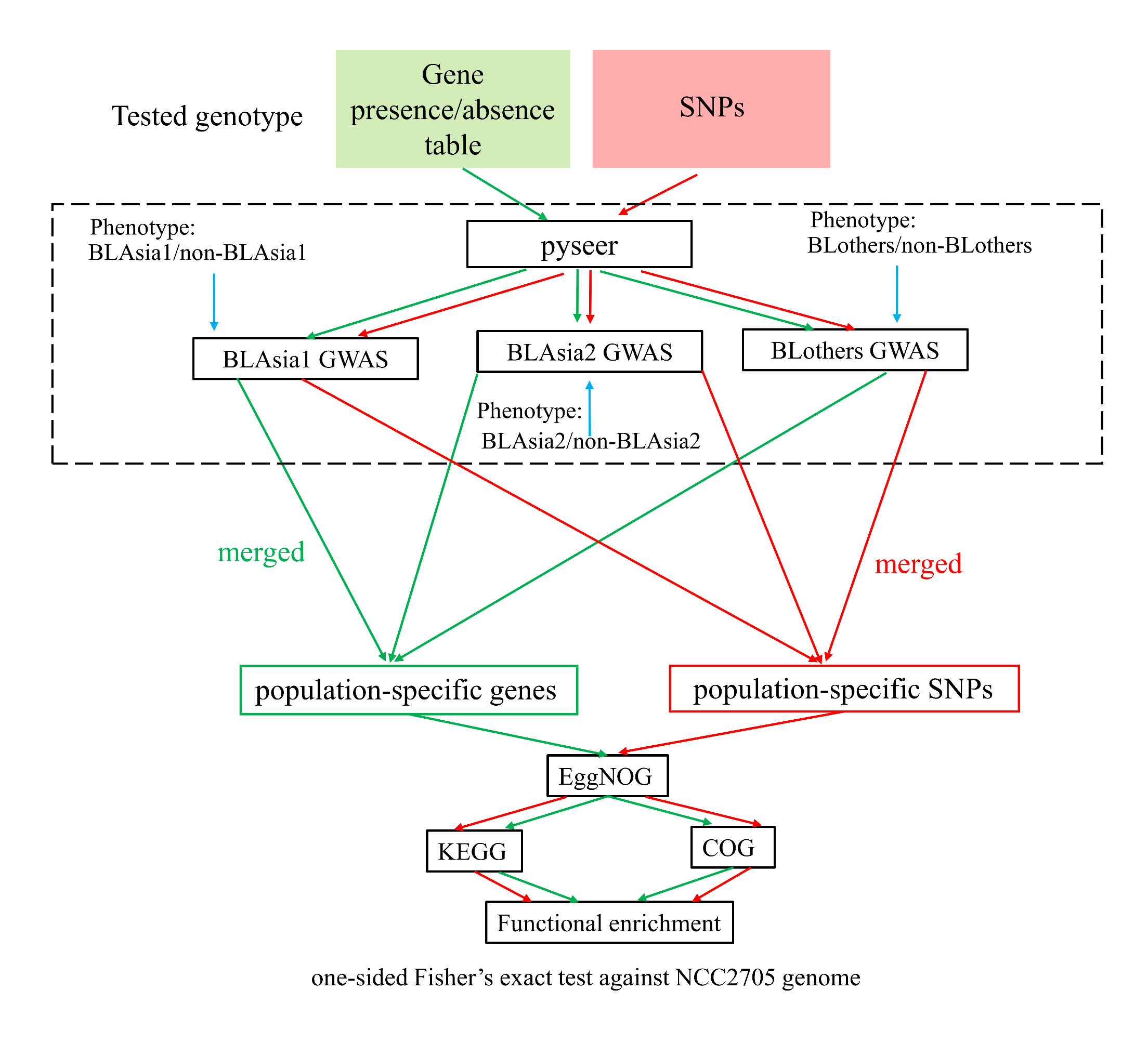


Figure S7. The flow chart of approaches used for identification of population-specific genes/SNPs.


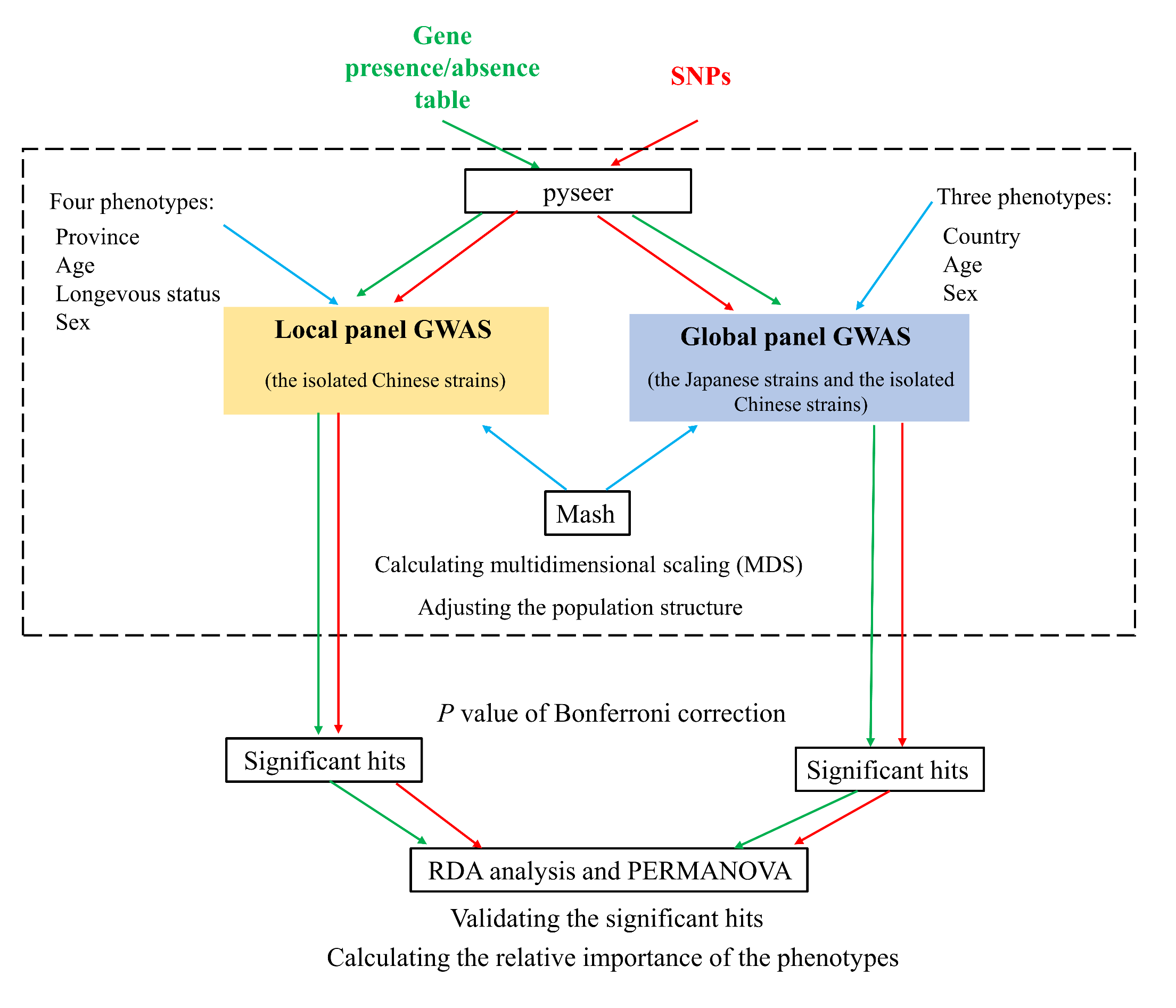


Figure S8. The flow chart of approaches used for phenotype association mapping.


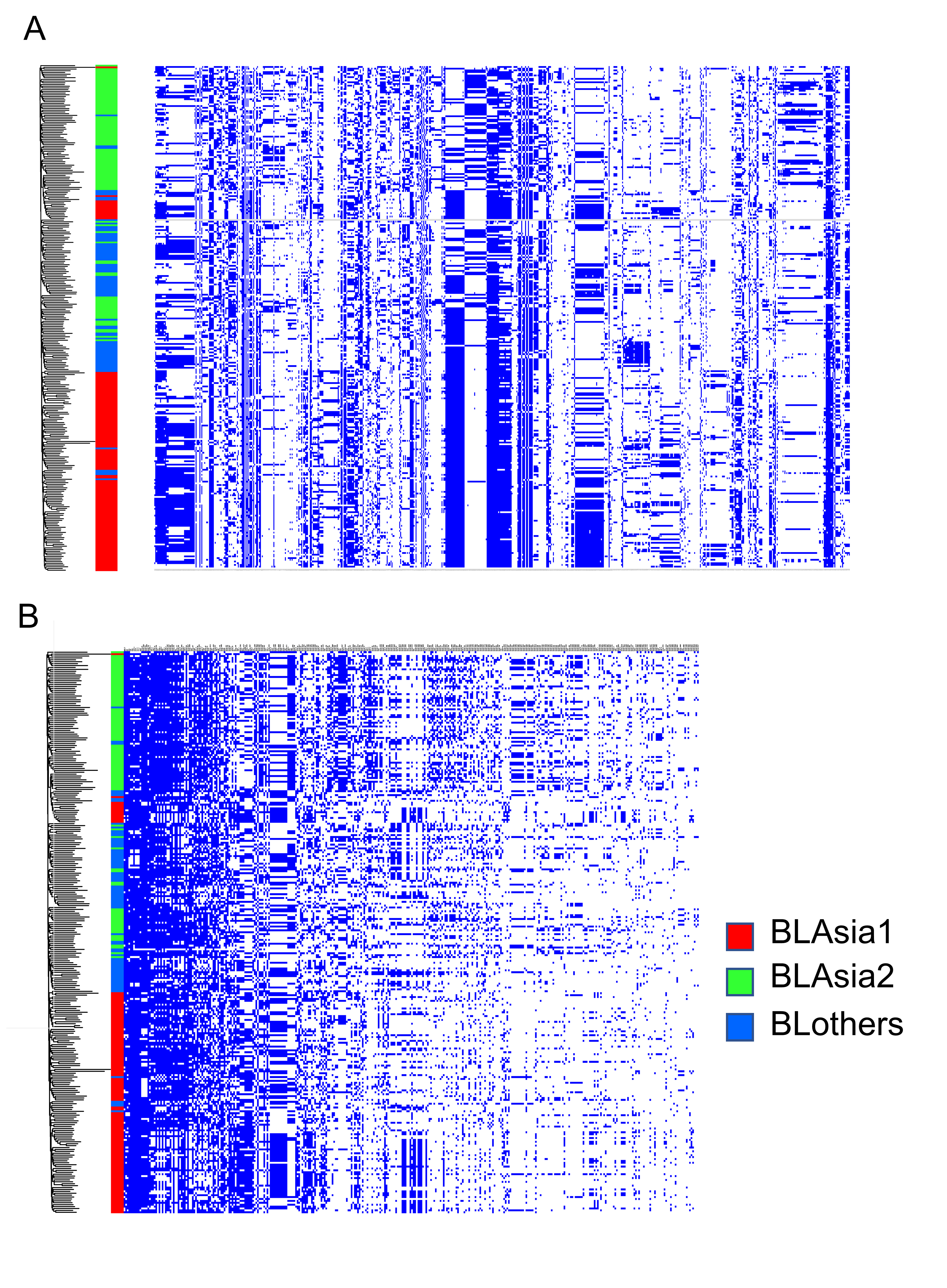


Figure S9. Population-specific variants are not driven solely by the population clonal structure. (A) Population-specific single nucleotide polymorphisms (SNPs) detected in 295 representative strains categorized into the BLAsia1 (n=124), BLAsia2 (n=97) and BLothers (n=74) groups. (B) Population-specific genes. Heatmaps are plotted against phylogeny (left), and the absence of each specific gene or an allele status identical to the reference genome is indicated in white, whereas the presence of each individual gene or an allele status different from the reference genome is indicated in blue.


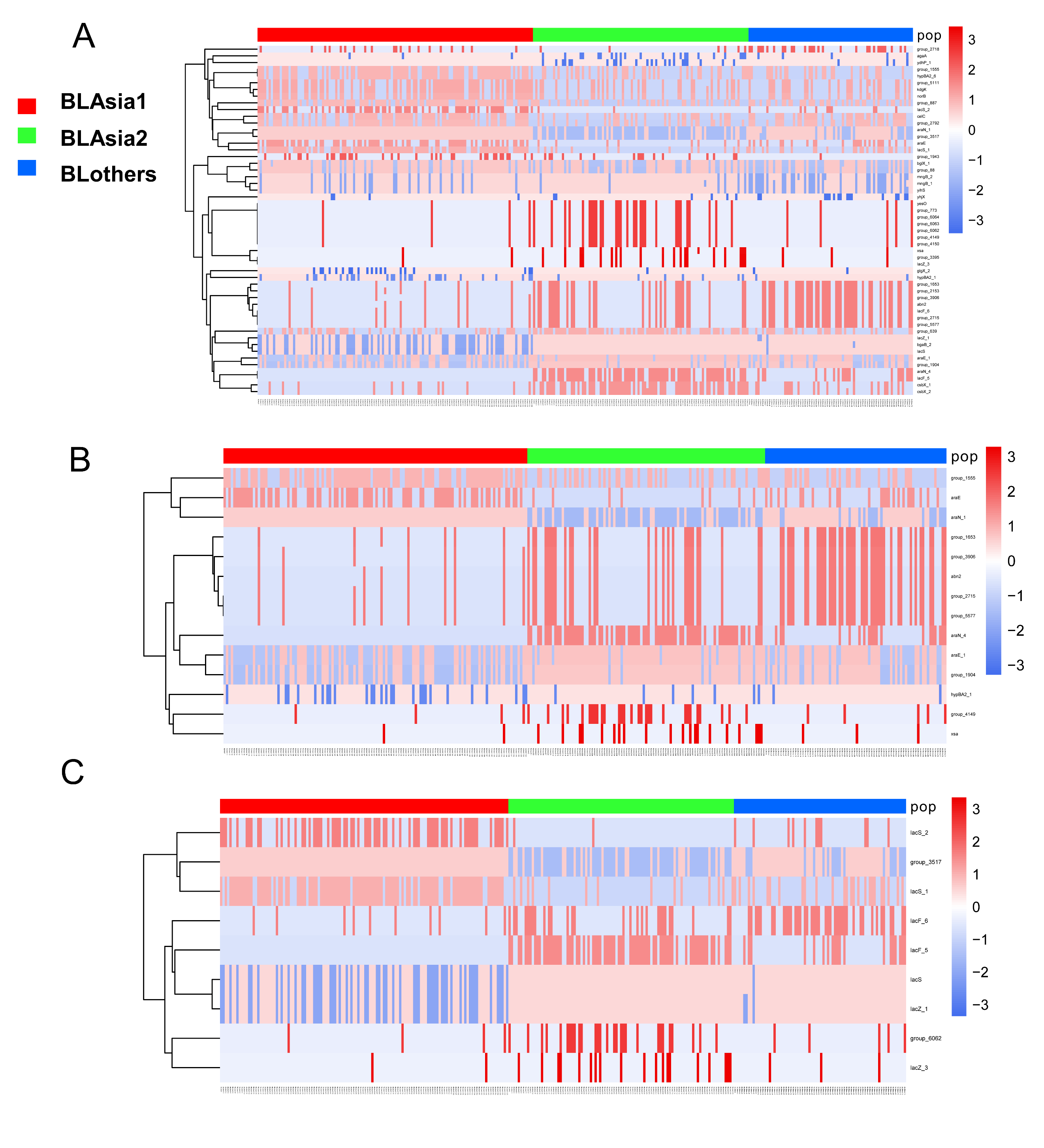


Figure S10. Genes involved in carbohydrate transport and metabolism (A) were significantly differentiated between *B. longum* populations, particularly those involved in arabinose (B) and lactose metabolism (C). Each column in the heatmaps represents a strain, whereas each row indicates a population-specific gene. The bar at the top is coloured by the populations that correspond to the individual strains. The gene presence and absence data were plotted and scaled for each row.


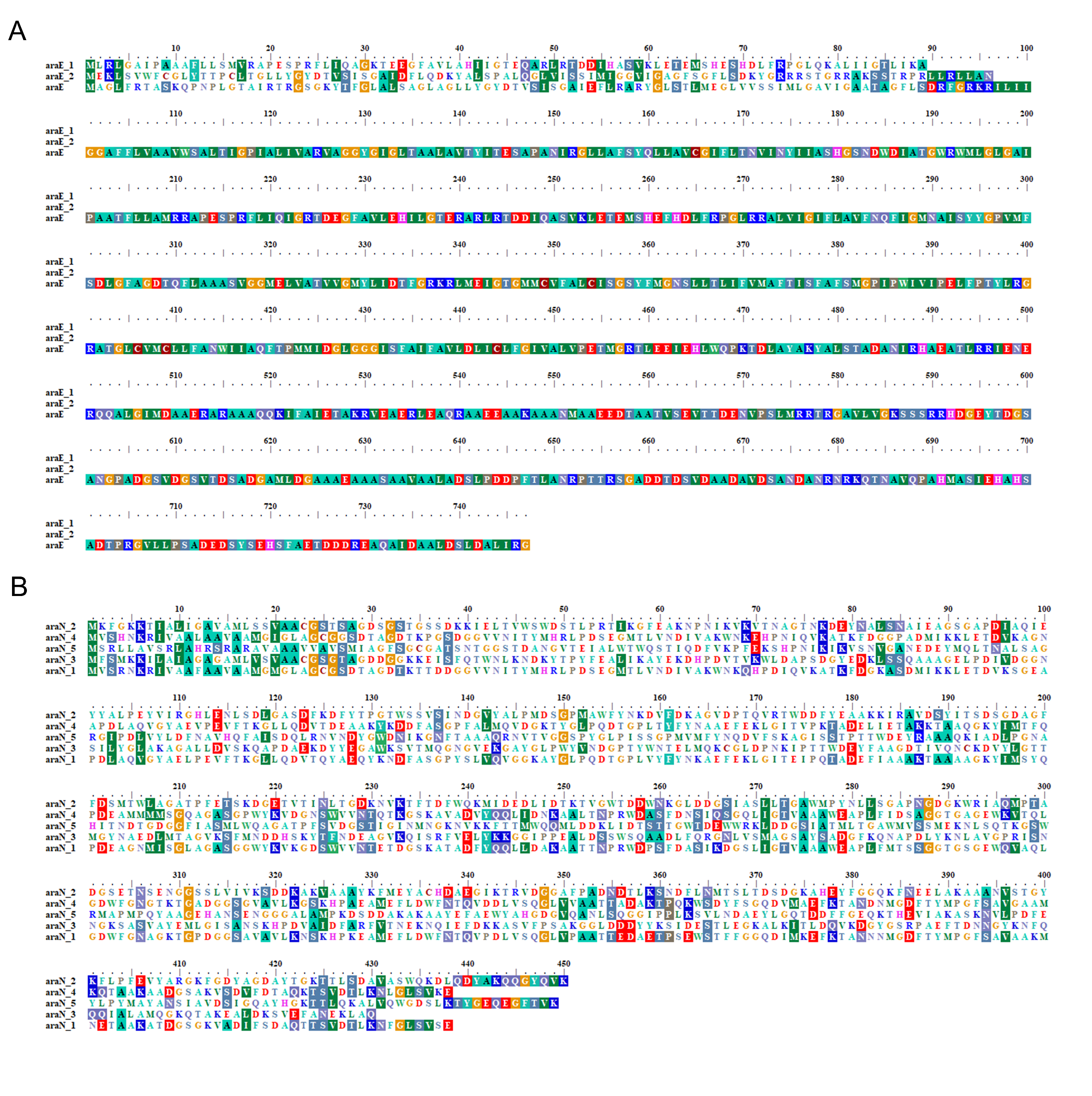


Figure S11. Sequence alignments for distinct paralogs of the specific genes araE(A) and araN(B). Representative sequences were used with priority selection from the complete genomes.


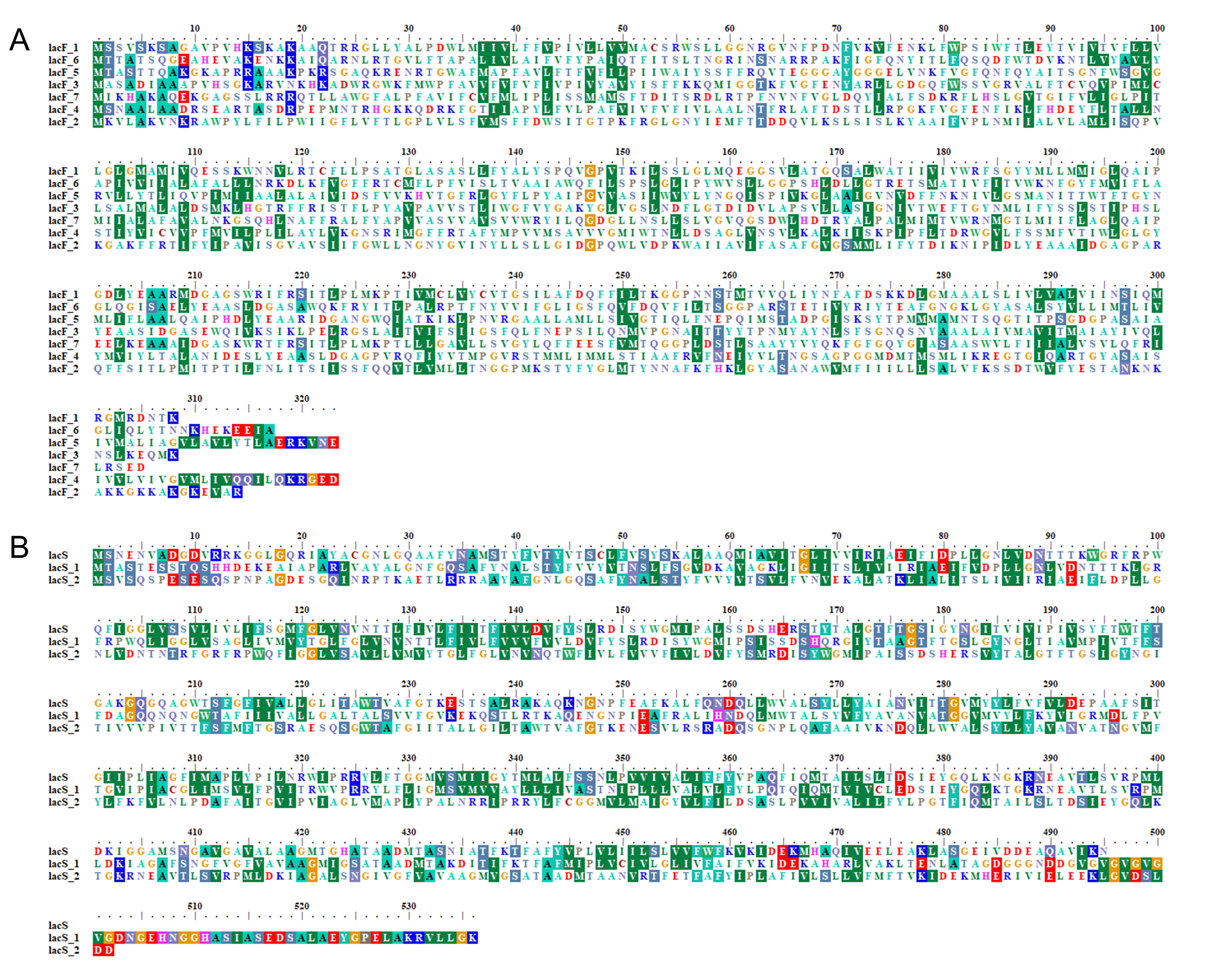


Figure S12. Sequence alignments for distinct paralogs of the specific genes, lacF(A) and lacS(B). Representative sequences were used with priority selection from the complete genomes.


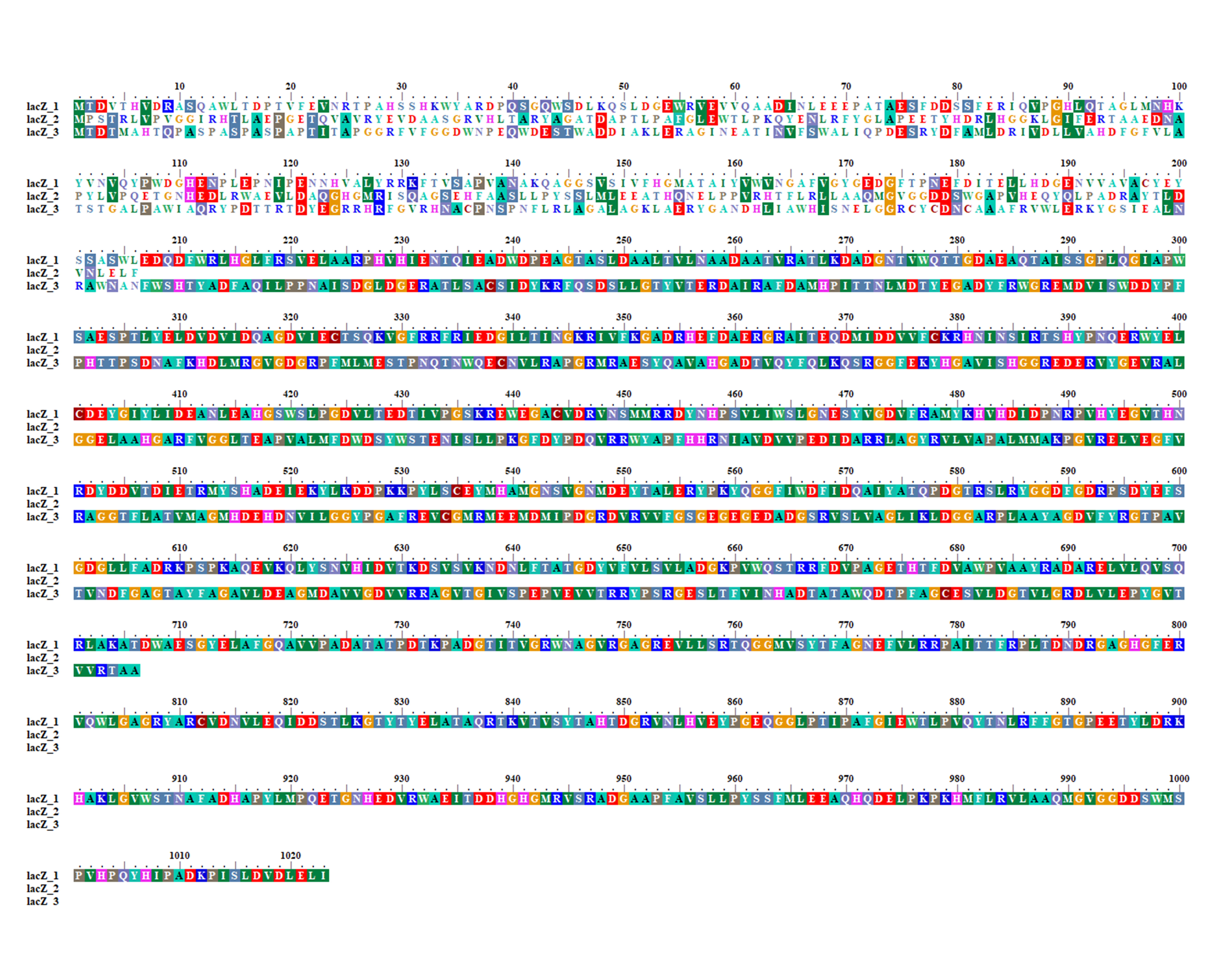


Figure S13. Sequence alignments for distinct paralogs of lacZ. Representative sequences were used with priority selection from the complete genomes.


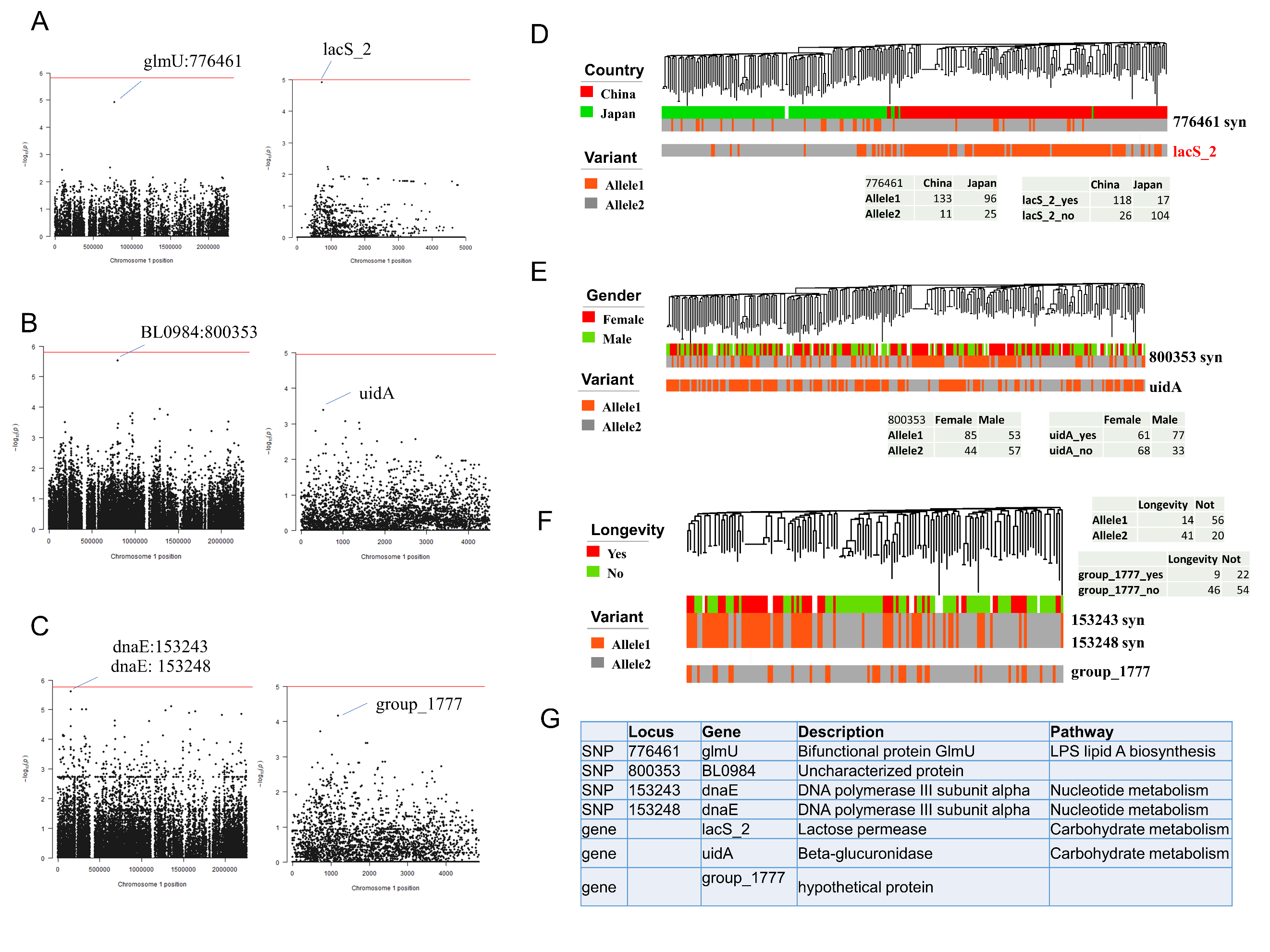


Figure S14. Lack of significant variants for the associations between host factors (country, sex, longevous district status) and the overall *B. longum* genotypes. Manhattan plot of GWAS results for the associations between host factors, including country (A), sex (B), and longevous district status (C), and the *B. longum* genomic profiles, based on SNPs in the core genome (left) and genes (right). The significant threshold is indicated by a horizontal red line and was defined using the Bonferroni correction with a required *P*-value of 0.05 divided by the number of variants. The distributions of selected variants against the phylogeny and host factors, including country (D), sex (E) and longevous district status (F). Annotations of the selected variants (G).


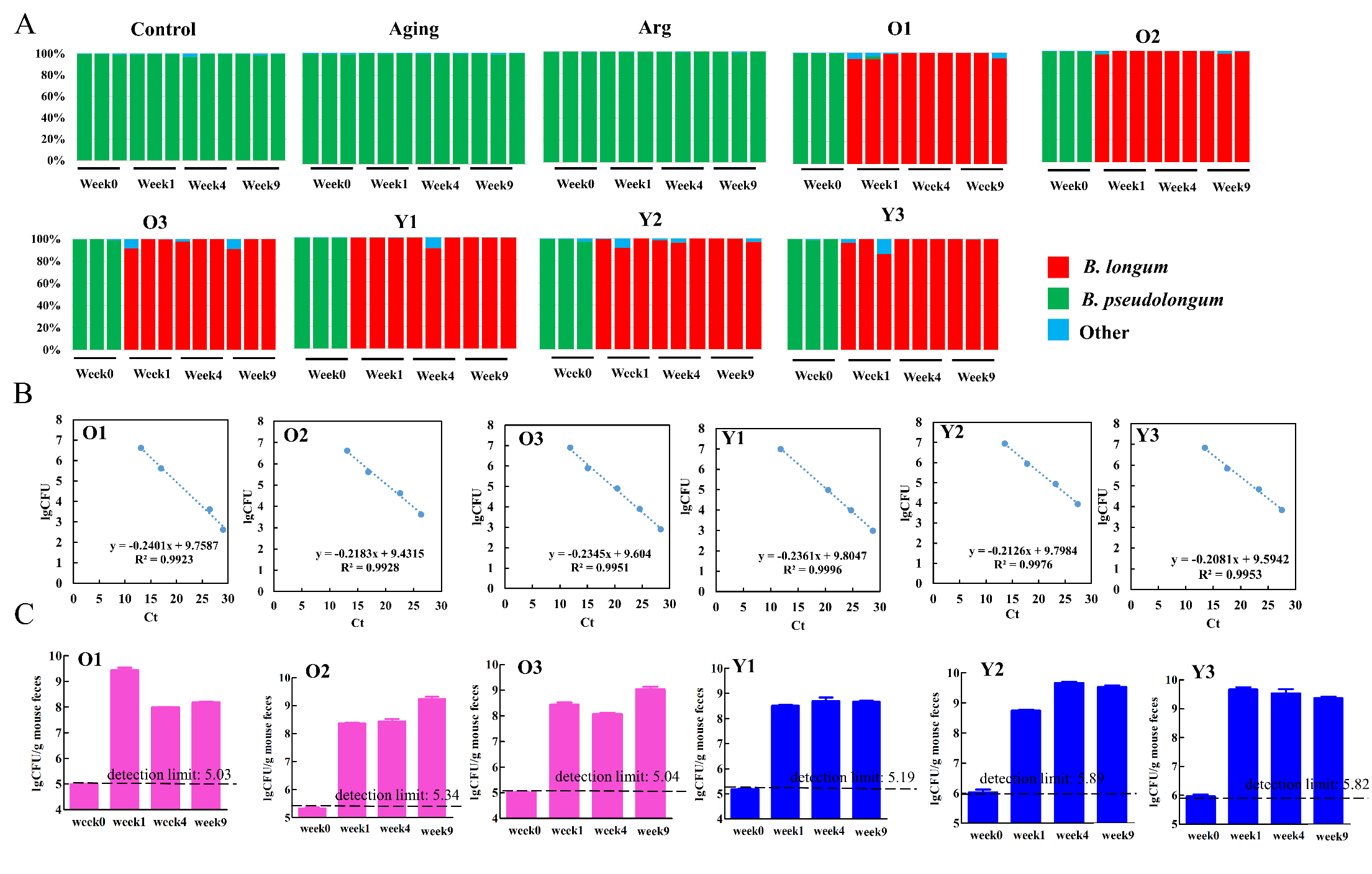


Figure S15. Each of the six administrated *B. longum* strains successfully engrafted in the gut of mice as detected by *groEL* gene based bifidobacterial profiling (A) and quantitative real-time PCR (B and C). (A) Fecal bifidobacterial composition of mice in nine experimental groups at the baseline, the week 1, the week 4, and the week 9. Each column indicates one fecal sample form an independent mouse. (B) Absolute quantitative standard curves for each of the target *B. longum* strains. (C) Absolute cell numbers of each of the *B. longum* strains in mouse fecal samples as determined by qPCR.


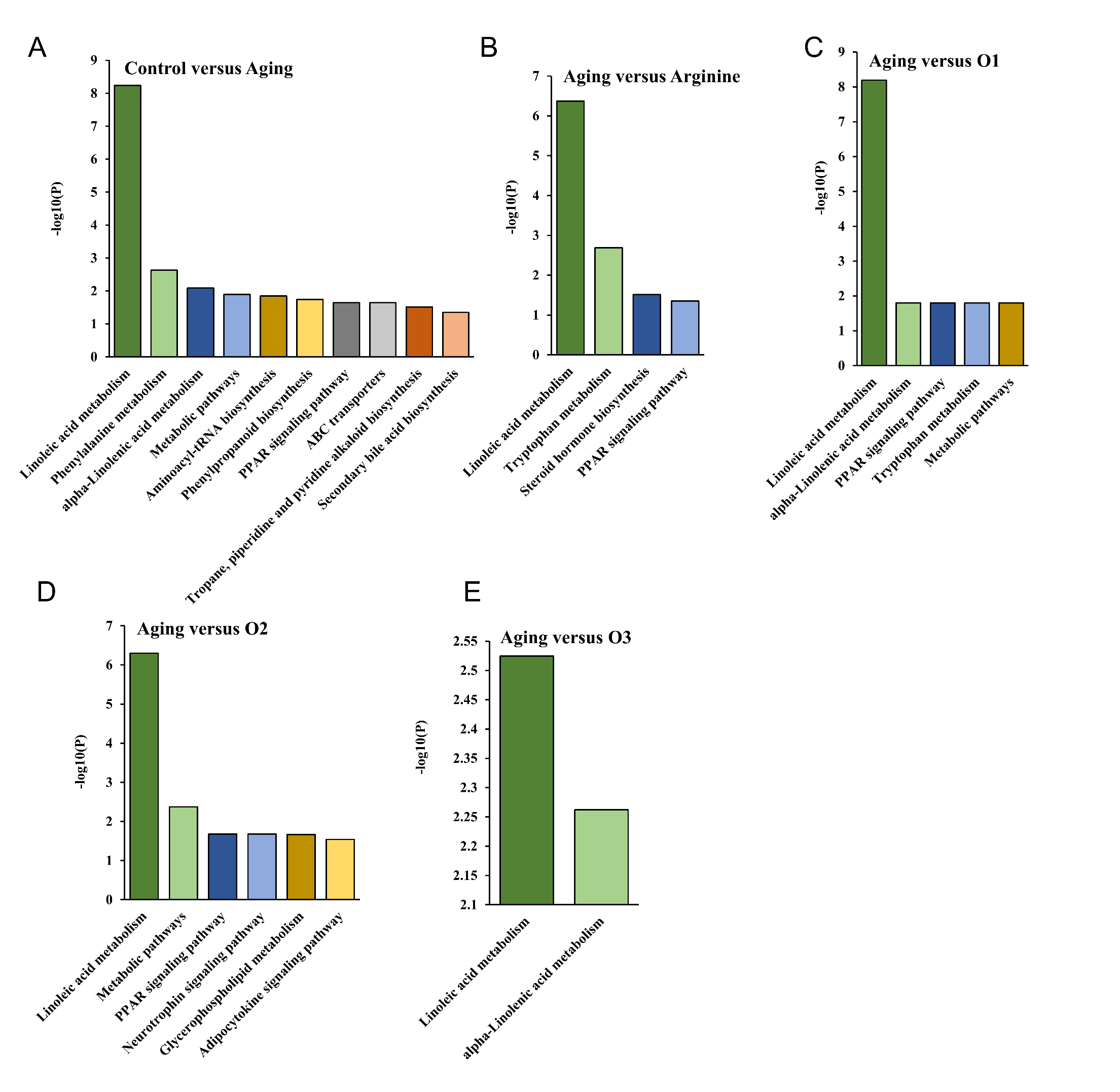


Figure S16. Significant differences in faecal metabolite pathways between treatments. The pathway enrichment analysis was conducted using metabolites with known Kyoto Encyclopaedia of Genes and Genomes (KEGG) IDs and searched against full the KEGG database at a significance threshold of FDR-adjusted *P* of less than 0.05.

**Supplementary references**

1. Lu J, Zheng Y-l, Luo L, Wu D-m, Sun D-x and Feng Y-j. Quercetin reverses D-galactose induced neurotoxicity in mouse brain. Behavioural brain research 2006; 171: 251-260.

2. Lu J, Zheng Y-l, Wu D-m, Sun D-x, Shan Q and Fan S-h. Trace amounts of copper induce neurotoxicity in the cholesterol‐fed mice through apoptosis. FEBS letters 2006; 580: 6730-6740.

3. Lu J, Zheng Y-L, Wu D-M, Luo L, Sun D-X and Shan Q. Ursolic acid ameliorates cognition deficits and attenuates oxidative damage in the brain of senescent mice induced by D-galactose. Biochemical pharmacology 2007; 74: 1078-1090.

4. Zhou Y, Dong Y, Xu Q, He Y, Tian S, Zhu S, et al. Mussel oligopeptides ameliorate cognition deficit and attenuate brain senescence in D-galactose-induced aging mice. Food and chemical toxicology 2013; 59: 412-420.

5. Ali T, Badshah H, Kim TH and Kim MO. Melatonin attenuates D‐galactose‐induced memory impairment, neuroinflammation and neurodegeneration via RAGE/NF‐KB/JNK signaling pathway in aging mouse model. Journal of pineal research 2015; 58: 71-85.

6. Rinne MM, Gueimonde M, Kalliomäki M, Hoppu U, Salminen SJ and Isolauri E. Similar bifidogenic effects of prebiotic-supplemented partially hydrolyzed infant formula and breastfeeding on infant gut microbiota. FEMS Immunology & Medical Microbiology 2005; 43: 59-65.

7. Maldonado-Gómez MX, Martínez I, Bottacini F, O’Callaghan A, Ventura M, van Sinderen D, et al. Stable engraftment of Bifidobacterium longum AH1206 in the human gut depends on individualized features of the resident microbiome. Cell host & microbe 2016; 20: 515-526.

8. Duranti S, Lugli GA, Mancabelli L, Armanini F, Turroni F, James K, et al. Maternal inheritance of bifidobacterial communities and bifidophages in infants through vertical transmission. Microbiome 2017; 5: 66.
